# Longitudinal associations between parent–child attachment and prosocial behaviour: the roles of empathy and gratitude

**DOI:** 10.3389/fpsyg.2026.1970907

**Published:** 2026-10-21

**Authors:** Xiaoxue Chen, Jia Xu, Anni Chen, Leigang Zhang

**Affiliations:** 1 School of Education and Psychology, Shaoxing University, Shaoxing, China; 2 Shaoxing Changcheng Middle School, Shaoxing, China

**Keywords:** adolescence, attachment, cross-lagged panel model, empathy, gratitude, longitudinal design, measurement invariance, prosocial behaviour

## Abstract

**Background:**

Parent–child attachment is assumed to support adolescent prosocial behaviour through empathy and gratitude, but most evidence is cross-sectional and rarely separates stable between-person differences from within-person change. This study examined whether longitudinal associations among these constructs match the proposed sequential account and whether they hold within persons.

**Methods:**

Chinese junior secondary students (*N* = 557 linked across three waves) completed an identical self-report battery on all four constructs. Longitudinal measurement invariance was tested for each construct. A random-intercept cross-lagged panel model (RI-CLPM) separated between-person from within-person variance, and a latent sequential indirect-effect model with autoregressive controls was estimated by full-information maximum likelihood with 5,000 bias-corrected bootstrap resamples. Two prespecified robustness analyses varied the placement of gratitude and excluded outliers.

**Results:**

Metric invariance held for all constructs; full scalar invariance failed for prosocial behaviour (ΔRMSEA = 0.021). Random intercepts held 64.5 to 70.9% of total variance, and no hypothesised within-person cross-lagged path was significant (attachment to empathy β = 0.076, *p* = 0.561; empathy to gratitude β = 0.140, *p* = 0.160; gratitude to prosocial behaviour β = −0.043, *p* = 0.694). In the latent model (CFI = 0.974, RMSEA = 0.046), attachment predicted empathy (B = 0.100, 95% CI [0.032, 0.163]), empathy predicted gratitude (B = 0.171 [0.015, 0.329]) and gratitude predicted prosocial behaviour (B = 0.376 [0.241, 0.545]). The sequential indirect association was small (B = 0.0064 [0.0010, 0.0202]; 5.8% of the total indirect association), was not reproduced when gratitude was placed at Wave 2, and its empathy-to-gratitude component lost significance after outliers were excluded. The two prospective two-link within-person indirect products identifiable in the RI-CLPM were also not distinguishable from zero.

**Conclusion:**

The data support stable longitudinal associations, particularly those involving gratitude, but only tentative evidence for a sequential pathway and none for a within-person developmental chain. A four-wave design placing each construct on a separate occasion is needed to test the mechanism.

## 1 Introduction

Prosocial behaviour, understood as voluntary action intended to benefit another person, is one of the developmental achievements that most clearly marks the transition from childhood into adolescence. Helping, sharing, comforting and cooperating predict peer acceptance, academic engagement and emotional wellbeing, and their absence forecasts a broad range of later difficulties. Longitudinal evidence links helping others to fewer internalising symptoms through the character strengths it cultivates (Padilla-Walker et al., 2020), and daily diary data from Chinese adolescents show that prosocial acts spill over into subjective wellbeing within the same person (Zhang W. et al., 2022). Prosocial tendencies neither rise smoothly nor stay flat across this period; they follow differentiated trajectories that depend on the target of the behaviour and on the context in which it is measured (Sweijen et al., 2025). Understanding what sustains prosociality during these years therefore has both theoretical and practical value.

Attachment theory supplies the most established account of why family relationships should matter here. Repeated experiences of sensitive care are internalised as working models of self and other, and a child who expects care to be available is freed from self-protective vigilance and becomes able to attend to the needs of others. Recent reappraisals of the framework note that the mechanisms connecting attachment to social competence remain incompletely specified (Thompson et al., 2022). The empirical picture is more uneven than the theory suggests. A systematic review of sixteen studies found inconsistent direct associations between attachment security and prosocial behaviour, with nine studies reporting significant links and seven not, whereas the association between attachment and empathy was considerably more reliable (Costa Martins et al., 2022). Parenting research reaches a compatible conclusion from a different angle: in a nationally representative sample of 5,317 adolescents, parental warmth and monitoring were related to prosocial behaviour through self-control and identification with family and friends rather than directly (Backman et al., 2024). The family is also not the only socialising context, since adolescents take on the prosocial tendencies of classmates they like (Chávez et al., 2025). Two implications follow. The direct attachment-to-prosociality path may be weak once intervening variables are modelled, and those intervening variables deserve as much theoretical attention as the predictor.

Two candidate intervening constructs have strong theoretical warrant. The first is empathy. The empathy-altruism hypothesis holds that empathic concern for a person in need produces motivation directed at that person’s welfare rather than at the observer’s own relief. Empathy is the most frequently tested intermediary between relationship quality and prosociality, and meta-analytic evidence supports that role in the peer domain (Li et al., 2025) and in relationships with several socialisation agents (Carrizales et al., 2023). Peer attachment predicted prosocial behaviour through empathy in a Spanish sample of 800 adolescents (Schoeps et al., 2020), and a model routing all attachment effects through empathic concern outperformed a direct-effects model in a South African sample (Profe et al., 2021). Empathy is not a single capacity, however. Affective sharing and cognitive understanding are distinguishable, with partly different correlates (Håkansson Eklund and Summer Meranius, 2021), distinct components carry associations with prosocial behaviour to different degrees (Chen et al., 2023), and the facets relate differently to reactive and proactive aggression (Palumbo and Latzman, 2021; Ritchie et al., 2022). Developmental work also indicates that empathy and prosocial behaviour follow partly separate trajectories across adolescence rather than moving in lockstep (Marshall et al., 2020). Empathy-based mediation also fails to replicate in every sample (Widyastuti and Savitri, 2023), and dispositional predictors of effortful and effortless helping diverge (Harris et al., 2023). Empathy is therefore best treated as an enabling condition rather than a sufficient cause of helping.

The second candidate is gratitude. The find-remind-and-bind account holds that gratitude functions to identify responsive relationship partners, bring existing bonds back to mind and strengthen them through reciprocal investment (Algoe, 2012). Gratitude on this account is relational rather than transactional, which makes the parent–child bond a natural site for it, and the broaden-and-build framework supplies the accumulation mechanism by which positive emotions widen momentary thought-action repertoires and build durable social resources (Fredrickson, 2001). Evidence from Chinese samples supports the attachment-to-gratitude link: in a three-wave study of 1,348 adolescents, attachment anxiety and avoidance negatively predicted gratitude six months later through perceived parental communal strength (Li et al., 2023), and gratitude mediated the association between attachment and life satisfaction in young adults (Lin, 2020). On the outcome side, gratitude predicts adolescent prosocial behaviour (Zhang, 2022), mediates the association between parental warmth and prosociality with school climate moderating the second half of the path (Luo et al., 2021; Zeng et al., 2023), and buffers the effect of bullying victimisation on self-injury (Wu et al., 2023). Intervention evidence adds a causal note, since expressed gratitude produces measurable wellbeing benefits relative to neutral comparison conditions (Kirca et al., 2023). Measurement work adds a caveat, because gratitude has cognitive and affective components that relate differently to outcomes (Zhang L. et al., 2022).

Taken together these strands suggest an ordered chain rather than two parallel routes. A secure bond makes the emotional states of others salient and safe to engage with, which supports empathy. Empathic attunement to what others give, and to what giving costs them, is a plausible precondition for recognising benefaction, which is what gratitude requires. Gratitude then motivates outward-directed action toward benefactors and, by generalisation, toward others. Empathy and gratitude jointly mediated the association between childhood maltreatment and prosocial behaviour in Chinese adolescents, which is the closest existing test of this ordering, but that design was cross-sectional and could not establish the sequence (Yu et al., 2020).

Three limitations recur across this literature. Most studies of empathy or gratitude as explanatory variables are cross-sectional. Longitudinal studies usually examine a single intermediary construct. Traditional cross-lagged analyses often do not separate stable between-person differences from within-person deviations, so a coefficient answers a between-person question while being described in within-person language (Hamaker et al., 2015; Orth et al., 2021). The closest precedent to the present design estimated both a traditional and a random-intercept cross-lagged panel model for self-compassion, gratitude and prosocial behaviour in Chinese adolescents, and found the gratitude-prosociality association between persons but not within them (Yang et al., 2021). What remains unresolved is whether the proposed associations persist when stable between-person differences are separated from within-person change, and when the temporal placement of empathy, gratitude and prosocial behaviour is varied across specifications.

The present study addresses that question on three fronts. First, it examines longitudinal associations consistent with the proposed attachment-empathy-gratitude-prosociality sequence across three measurement occasions, while recognising that a three-wave design cannot place four constructs on four distinct occasions. Second, it applies a random-intercept cross-lagged panel model to separate between-person differences from within-person change before any developmental claim is made. Third, it reports the decomposed and undecomposed estimates side by side rather than only the one that supports a sequential reading, and it tests whether the result is sensitive to measuring the second intermediary construct concurrently with the outcome. Four hypotheses were specified in advance.

H1. Parent-child attachment at Wave 1 is positively associated with empathy at Wave 2 after adjustment for Wave 1 empathy.

H2. Empathy at Wave 2 is positively associated with gratitude at Wave 3 after adjustment for Wave 1 gratitude and Wave 1 attachment.

H3a. In the temporally lagged two-step specification, the indirect association between Wave 1 attachment and Wave 3 prosocial behaviour through Wave 2 gratitude differs from zero.

H3b (exploratory). A sequential indirect association runs from Wave 1 attachment through Wave 2 empathy and Wave 3 gratitude to Wave 3 prosocial behaviour. Because gratitude and prosocial behaviour are measured concurrently at Wave 3, this hypothesis is treated as exploratory rather than as a fully temporally ordered test.

H4. After stable between-person differences are separated through random intercepts, the within-person cross-lagged coefficients corresponding to the proposed sequence are substantially attenuated relative to those from the traditional cross-lagged panel model.

## 2 Materials and methods

### 2.1 Study design, participants, and ethical approval

The study used a three-wave school-based panel design. Participants were students at a single junior secondary school in China who completed the same paper questionnaire, containing four instruments in a fixed order, on three successive occasions. The archived materials do not identify the city or province, and they do not preserve calendar dates or semester identifiers; consequently, the elapsed interval between waves cannot be reconstructed and all cross-lagged estimates must be interpreted without a known lag length.

The study was conducted in accordance with the Declaration of Helsinki. The research protocol was approved by the Ethics Committee of the Affiliated Hospital of Shaoxing University (Ethics Approval Number: 2023–105-01). Because all participants were minors, written informed consent was obtained from a parent or legal guardian of every participating adolescent, and written informed assent was obtained from each adolescent, before the first wave of data collection. Institutional permission for the school-based data collection was granted by the participating school. Participation was voluntary at every measurement occasion, adolescents were free to stop at any point without consequence, and responses were recorded against coded participant identifiers rather than names, so that no directly identifying information was held in the analysis file. These conditions govern the availability of the data, as set out in the Availability of Data and Materials statement.

The archived dataset contained 563 records at Wave 1, 558 at Wave 2 and 557 at Wave 3. Identifier matching showed 557 adolescents represented at all three waves; six Wave 1 records were absent at Wave 3, and Wave 2 contained one additional record outside the complete three-wave linkage. The longitudinal models therefore used the 557 linked adolescents. Of these, 475 had valid scores on all 12 wave-by-construct composites and 82 were missing at least one composite; the latter would have been lost under listwise deletion but were retained under full-information maximum likelihood (FIML). Descriptive and reliability analyses that summarise a single wave use all records available at that wave, which is why some wave-specific denominators differ from the longitudinal model N of 557.

The demographic section recorded gender, age band, grade level, sibling status and prior participation in voluntary activity. At Wave 1, 285 participants were coded as male and 246 as female, with 32 gender responses missing. The workbook comments identify the grade codes as first-, second- and third-year junior secondary. Grade level was concentrated in the first-year category (*n* = 490), with 42 participants in the second-year category and 31 missing; no third-year codes occur in the archived file. The panel is therefore drawn from a single dominant first-year cohort with a small second-year group. Gender and age band were retained as covariates in the latent models.

The archived records do not contain calendar dates or semester identifiers from which the spacing between occasions can be reconstructed, so the waves are treated as three successive measurement occasions and the lag-dependence of cross-lagged coefficients is acknowledged as a limitation. Each participant carried a stable identifier across waves, permitting exact record linkage without probabilistic matching, and administration conditions, item wording and response format were identical on all three occasions, which is the condition required for the invariance tests reported below. One indirect indication of elapsed time comes from the age band: among the 514 linked participants supplying a band at both Wave 1 and Wave 3, 73.5% reported the same band, 17.1% a higher band and 9.3% a lower one, which is consistent with a total span short relative to the width of the age categories but does not identify the interval in months.

### 2.2 Measures

#### 2.2.1 Parent–child attachment

Attachment was assessed with 20 items arranged as ten parallel mother- and father-referenced content pairs, rated on a five-point scale, with eight items reverse-scored. The wording corresponds to domains represented in the Inventory of Parent and Peer Attachment (Armsden and Greenberg, 1987); the archived materials do not identify a published 10-item parent short form or preserve documentation of the original item-selection, adaptation or Chinese translation procedure, so the measure is described as a project-specific adaptation rather than a formally validated short form. A descriptive structural check showed a dominant common component accounting for 41.3%, 42.7 and 44.6% of item variance at Waves 1 to 3. Mother- and father-referenced composites correlated at r = 0.820, 0.798 and 0.806 across waves, and each parent-specific composite correlated above 0.945 with the combined score, which supports the combined index used here; parent-specific effects remain outside the scope of the study.

The archived questionnaire contains a wording anomaly at item D14, whose administered string duplicates D13 and refers to the mother although the alternating structure of the surrounding items places it in the father-referent position. The two variables were not duplicates at the response level, differing in 10.2 to 15.5% of cases and correlating at r = 0.873, 0.815 and 0.838 across waves. Excluding D14 produced attachment alpha coefficients of 0.920, 0.925 and 0.928 against 0.922, 0.928 and 0.931 for the 20-item score, full and D14-excluded scores correlated above 0.997 at every wave, and the sequential product changed only from 0.00780 to 0.00771. D14 was therefore retained to preserve the administered questionnaire.

#### 2.2.2 Empathy

Empathy was measured with the 20-item Basic Empathy Scale (Jolliffe and Farrington, 2006), for which a Chinese validation is available (Geng et al., 2012). Eleven items index affective empathy and nine index cognitive empathy; items were rated on a five-point scale and seven were reverse-scored. One item that is reverse-keyed in the original was administered in positively worded form and scored accordingly. The two components correlated at r = 0.349, 0.427 and 0.473 across waves, indicating related but non-equivalent dimensions. The primary model retained the total score because the hypothesised sequence concerns broad empathic responsiveness and because a single broad score permits direct comparison with the other focal constructs across the three-wave panel. However, aggregation could obscure facet-specific associations. We therefore added a *post hoc* observed-score sensitivity analysis that examined affective and cognitive empathy separately; those results are reported in Section 3.4.1. The broad empathy result is not interpreted as evidence that the two facets contribute equally.

#### 2.2.3 Gratitude

Gratitude was assessed with the six-item Gratitude Questionnaire (McCullough et al., 2002), rated on a five-point scale, with two items reverse-scored.

#### 2.2.4 Prosocial behaviour

Prosocial behaviour was assessed with the 15-item self-report inventory included in the project questionnaire battery. Items describe concrete acts across school, family and community settings, are all positively worded, are rated on a five-point scale, and none is reverse-scored; an English translation of the full administered set is reproduced in Supplementary Table S1. The archived materials do not name a published source scale and contain no documentation of item development, expert review, pilot testing or external criterion validation, so these procedures are not claimed retrospectively. The internal structure was therefore examined directly in the study data. A dominant one-factor solution was observed at each occasion, accounting for 53.6%, 53.8 and 65.6% of item variance, with absolute loadings from 0.529 to 0.857 across waves. As an internal convergent check, prosocial behaviour correlated positively with attachment, empathy and gratitude at every occasion (r = 0.295 to 0.581, all *p* < 0.001). As an additional *post hoc* criterion-related check, the demographic item on participation in school or community public-welfare activities (1 = frequent, 2 = occasional, 3 = never) was compared with the prosocial score at each wave by one-way analysis of variance and Spearman rank correlation. Because this criterion was also self-reported, it is treated as supportive within-sample evidence rather than independent external validation.

#### 2.2.5 Reliability and common-method diagnostics

Internal consistency was computed from the study data at every wave. Cronbach’s alpha was calculated from the item variances and the variance of the scale total, and McDonald’s omega from the loadings of a one-factor solution fitted to the item correlation matrix by iterated principal axis extraction. Omega is reported alongside alpha because alpha assumes that every item loads equally on the underlying factor, an assumption that rarely holds for the multi-facet empathy and attachment measures used here. Coefficients appear in Table 1. Affective and cognitive empathy subscale values, which are not shown there, ranged from 0.743 to 0.785 and 0.795 to 0.864 for alpha across the three waves. Item-level heterotrait-monotrait ratios were below the conservative 0.85 criterion for all construct pairs, with maxima of 0.611, 0.665 and 0.812 at Waves 1 to 3. As a descriptive common-method diagnostic, the first unrotated component across all focal items accounted for 24.0%, 26.2 and 30.6% of variance across the three waves. This single-factor result is not treated as a statistical control for common-method variance and cannot rule it out. The archive contains no marker variable, alternate informant, or alternate response format that would permit a stronger design-based control, and fitting an unmeasured method factor would require identification constraints not supported by the archived measurement design. Stable response style or social desirability may therefore contribute to the random-intercept correlations and to associations in the undecomposed latent model. The RI-CLPM removes time-invariant between-person covariance before estimating within-person paths, but it does not eliminate possible time-varying shared-method effects.

**Table 1:** Descriptive statistics and internal consistency for study variables across three waves.

Variable	*n*	M	SD	Skewness	Kurtosis	α	ω
Parent–child attachment W1	532	3.818	0.749	−0.255	−0.503	0.922	0.921
Parent–child attachment W2	504	3.814	0.736	−0.162	−0.727	0.927	0.928
Parent–child attachment W3	536	3.776	0.746	0.098	−0.703	0.931	0.929
Empathy W1	532	3.467	0.522	0.211	0.533	0.812	0.795
Empathy W2	504	3.485	0.555	0.131	0.294	0.852	0.846
Empathy W3	536	3.515	0.568	0.241	0.232	0.865	0.859
Gratitude W1	531	3.691	0.766	−0.095	−0.212	0.760	0.775
Gratitude W2	504	3.673	0.844	−0.208	−0.275	0.809	0.826
Gratitude W3	536	3.667	0.818	−0.116	−0.224	0.783	0.801
Prosocial behaviour W1	532	3.914	0.760	−0.430	0.444	0.937	0.938
Prosocial behaviour W2	504	3.855	0.748	−0.259	0.090	0.938	0.939
Prosocial behaviour W3	536	3.940	0.805	−0.590	0.623	0.962	0.963

All variables are item means on a one-to-five response scale. α = Cronbach’s alpha; ω = McDonald’s omega from a one-factor solution. W1, W2, and W3 denote the three waves. Reliability coefficients use all valid responses available at each wave.

### 2.3 Data preparation

The three wave-specific worksheets were read with a two-row header structure, item labels were normalised to instrument codes, and response fields were coerced to numeric with non-numeric entries set to missing. All item responses fell inside the intended one-to-five range at every wave, so no out-of-range recoding was required, and reverse-keyed items were recoded before any aggregation. Scale scores were formed as item means, computed only when a participant answered at least 60% of the items in that scale. For the latent models, three item parcels were built per construct under a domain-representative allocation, so that each parcel drew proportionally from the affective and cognitive facets of empathy and from the mother- and father-referent halves of the attachment measure. Parcel membership was fixed on the Wave 1 data and applied unchanged to Waves 2 and 3, which is the condition required for meaningful invariance testing (Little et al., 2002). The full parcel construction, including item pools, parcel sizes and balancing constraints, is given in Supplementary Table S2.

Item-level missingness was 5.8% at Wave 1, 9.8% at Wave 2 and 3.9% at Wave 3, and was clustered rather than spread uniformly across items. Thirty-one Wave 1 records, 54 Wave 2 records and 21 Wave 3 records yielded no valid score for any construct under the 60% completion rule, and at Wave 2 52 of these were completely blank across the 61 construct items, so the pattern reflects within-session non-completion by a small group rather than item-by-item refusal. Little’s test on the 12 wave-by-construct composites was significant, χ^2^(47) = 76.63, *p* = 0.004, so missing completely at random was rejected (Little, 1988). Rejecting that hypothesis does not establish that data are missing at random, which cannot be verified from observed data alone. Baseline comparisons showed no significant differences on the four Wave 1 construct scores between complete and non-complete cases (Supplementary Table S3), age band was unrelated to retention, χ^2^(3) = 1.89, *p* = 0.595, and the gender comparison was borderline under the continuity-corrected test, χ^2^(1) = 3.77, *p* = 0.052. Checks of grade, sibling status and prior volunteering likewise showed no significant association with retention. FIML was therefore used under a missing-at-random assumption conditional on the observed information in the models, rather than because that assumption had been established empirically (Enders, 2022). Under listwise deletion, 82 of the 557 linked participants would have been removed, which is 14.7% of the panel. No formal analysis of missingness not at random was available in the archived workflow, so the estimates should be read as conditional on the stated assumption.

Multivariate outliers were screened by Mahalanobis distance across the 12 composites among the 475 participants with complete data on all of them. Against a conservative χ^2^(12) criterion at *p* < 0.001, the critical value was 32.91 and 26 cases exceeded it, that is 5.47% of those screened, with a maximum distance of 87.89. Inspection showed internally consistent response profiles rather than data errors, so they were retained in the primary analyses and removed only in the prespecified sensitivity analysis, which left N = 531 after the 26 identified participants were dropped from the linked panel.

### 2.4 Statistical analysis

Data preparation was carried out in Python 3 and all models were estimated in R with the lavaan package (Rosseel, 2012) under FIML. Model fit was judged against conventional criteria: comparative fit index (CFI) and Tucker-Lewis index (TLI) at or above 0.95 for good and 0.90 for acceptable fit, root mean square error of approximation (RMSEA) at or below 0.06 for good and 0.08 for acceptable fit, and standardised root mean square residual (SRMR) at or below 0.08 (Hu and Bentler, 1999). The chi-square statistic is reported but not used as the sole basis for rejecting a model, since it is sensitive to sample size. Analyses proceeded through four ordered stages; the original modelling pipeline, together with the lavaan model syntax and data-preparation code, is provided in Supplementary Table S4. The additional *post hoc* checks described below are reported separately from that original pipeline.

*Stage 1: longitudinal measurement invariance*. Comparing constructs across time requires an equivalent measurement model on each occasion. For each construct a three-factor model was specified with one latent variable per wave and three parcel indicators each, with residual covariances freed for matching parcels across waves. Identification used the marker-variable approach, fixing the first loading to one and the first intercept to zero at each wave while freeing the latent means. Three nested models were compared: configural, with all loadings and intercepts free; metric, with loadings constrained to equality; and scalar, with intercepts additionally constrained. Because the likelihood ratio test is oversensitive at this sample size, invariance was judged primarily on change in fit, with an absolute change in CFI no greater than 0.010 and an increase in RMSEA no greater than 0.015 taken as evidence that a constraint held (Cheung and Rensvold, 2002; Chen, 2007). The measurement model is given in Equation 1.

$$x_{i,j,t}=\tau_{j,t}+\lambda_{j,t}\eta_{i,t}+\varepsilon_{i,j,t}$$ (1)

In Equation 1, x with subscripts i, j and t is the observed score of adolescent i on parcel j at wave t, where i runs from 1 to 557 and both j and t run from 1 to 3; τ is the intercept of parcel j at wave t; λ is the loading of parcel j at wave t; η is the latent construct score of adolescent i at wave t; and ε is the parcel-specific residual, assumed to have expectation zero and to be uncorrelated with the latent factor. Residuals of the same parcel are allowed to covary across waves because identical wording is presented on each occasion. Metric invariance imposes equality of λ across waves for each parcel, and scalar invariance imposes the same equality on τ.

*Stage 2: random-intercept cross-lagged panel model*. The traditional cross-lagged panel model regresses observed scores at one occasion on observed scores at the previous occasion, so its coefficients blend two conceptually distinct questions: whether adolescents who are generally higher on one construct are generally higher on another, and whether an adolescent who is temporarily above their own usual level on one construct is subsequently above their own usual level on another. The RI-CLPM resolves this by adding a time-invariant latent factor per construct with all loadings fixed to one, so that the cross-lagged structure is estimated among the residual within-person deviations (Hamaker et al., 2015; Mulder and Hamaker, 2021). Methodological reviews argue that the undecomposed model is rarely the right choice for the questions researchers actually ask (Lucas, 2023), and that the within-person and between-person distinction is informative about causal structure without settling it (Rohrer and Murayama, 2023). The decomposition is given in Equation 2 and the within-person dynamics in Equation 3.

$$y_{i,t}=\mu_{t}+\kappa_{i}+w_{i,t}$$ (2)

$$w_{i,t}=B_{t}w_{i,t-1}+\zeta_{i,t}$$ (3)

In Equation 2, y is the observed composite score of adolescent i at wave t; μ is the grand mean of that construct at wave t, which absorbs average change over time; κ is the adolescent-specific random intercept, a time-invariant factor with all three loadings fixed to one that captures how far adolescent i sits above or below the sample as a stable trait; and w is the remaining within-person deviation, that is how far adolescent i sits above or below their own usual level on that occasion. Random intercepts may covary with one another, which is where between-person association is captured, and are constrained orthogonal to the within-person components at the first occasion. Equation 3 applies for t equal to 2 and 3, giving one transition from Wave 1 to Wave 2 and one from Wave 2 to Wave 3. In Equation 3, w is the four-element vector of within-person deviations for attachment, empathy, gratitude and prosocial behaviour; B is the four-by-four coefficient matrix for the transition into wave t, whose diagonal entries are within-person autoregressive effects and whose twelve off-diagonal entries are within-person cross-lagged effects; and ζ is the vector of within-person innovations at wave t, whose elements may covary within each occasion but not across occasions. Thirty-two coefficients are estimated in total. The traditional model was estimated on the same composites so that the two can be compared on fit and on substantive conclusions. As a *post hoc* decomposition-based mediation check, we also formed the two prospective indirect products that can be identified within the three-wave RI-CLPM while ending in Wave 3 prosocial behaviour: attachment W1 to empathy W2 to prosocial behaviour W3, and attachment W1 to gratitude W2 to prosocial behaviour W3. A fully prospective four-variable attachment-empathy-gratitude-prosocial indirect effect is not identifiable with three waves because it would require a fourth occasion. Standard errors for the two identifiable products were obtained by the delta method from the RI-CLPM parameter-covariance matrix, and 95% Wald confidence intervals are reported.

*Stage 3: latent sequential indirect-effect model*. The indirect-effect model was estimated in a latent framework using the same parcel indicators, with loadings constrained to equality across waves in line with the metric invariance result and residual covariances freed for matching parcels. The predictor was attachment at Wave 1, the first intervening construct empathy at Wave 2, the second gratitude at Wave 3, and the outcome prosocial behaviour at Wave 3. Wave 1 latent scores for empathy, gratitude and prosocial behaviour entered as autoregressive controls so that every path is estimated on change relative to baseline, and gender and age band were included as covariates. In conventional notation, a1 is the attachment-to-empathy path, a2 the attachment-to-gratitude path, d the empathy-to-gratitude path, b1 the empathy-to-prosocial path, b2 the gratitude-to-prosocial path and c prime the direct attachment-to-prosocial path. The quantities of interest are defined in Equation 4.

$$\theta_{\mathrm{seq}}=a_{1}db_{2},\theta_{\mathrm{ind}}=a_{1}b_{1}+a_{2}b_{2}+a_{1}db_{2},\theta_{\mathrm{tot}}=c^{\prime }+\theta_{\mathrm{ind}}$$ (4)

In Equation 4, θ with subscript seq is the sequential indirect association running from attachment through empathy and then gratitude to prosocial behaviour; θ with subscript ind is the total indirect association, the sum of the three indirect routes; and θ with subscript tot is the total association of attachment with prosocial behaviour. Inference on these products used the bias-corrected bootstrap with 5,000 resamples drawn with replacement from the analytic sample, each refitted under FIML (Efron, 1987; Preacher and Hayes, 2008); every resample converged. The bias-corrected interval adjusts the percentile endpoints for median bias as shown in Equation 5, where z with subscript zero is the bias-correction constant, Φ is the standard normal cumulative distribution function and Φ raised to minus one its inverse, F with subscript B is the proportion of bootstrap estimates falling below the full-sample point estimate, and α with subscripts one and two are the adjusted percentiles at which the limits are read from the ordered bootstrap distribution. When the bootstrap distribution is symmetric, z with subscript zero equals zero and the interval reduces to the ordinary percentile interval, so both are reported. Estimates are presented as model-implied associations rather than causal quantities (Rohrer et al., 2022).

$$z_{0}=\Phi^{-1}(F_{B}),\alpha_{1}=\Phi (2z_{0}+z_{\alpha /2}),\alpha_{2}=\Phi (2z_{0}+z_{1-\alpha /2})$$ (5)

The primary specification does not fully order all four constructs in time, because gratitude and prosocial behaviour are both measured at Wave 3, so the gratitude-to-prosocial coefficient is concurrent rather than lagged and the corresponding sequential product is interpreted as an exploratory model-implied association. The RI-CLPM and the latent indirect-effect model also address different estimands. The former estimates prospective associations among occasion-specific within-person deviations after stable between-person differences have been removed; the latter makes no such decomposition, so its coefficients contain information associated with both stable between-person covariance and longitudinal variation. A detectable indirect product in the latter therefore cannot be read as evidence for a within-person mechanism, and non-significant within-person paths do not mathematically invalidate it. The added RI-CLPM indirect-product checks provide the decomposition-based sensitivity analysis, whereas the latent sequential model is retained as a complementary model-implied association. The two analyses answer different questions and are interpreted separately.

*Stage 4: robustness*. Two robustness analyses were specified before the results were interpreted, and both are reported in full whether or not they agreed with the primary model. The first re-specified the model with gratitude measured at Wave 2 rather than Wave 3, so that the second intervening construct precedes the outcome; this model was estimated with maximum-likelihood intervals and was not bootstrapped, so its intervals are not directly comparable with the bias-corrected bootstrap intervals of the primary model. Neither three-wave specification places all four variables on separate occasions, since empathy and gratitude are then concurrent at Wave 2; a minimum four-wave design is required for that test. The second re-estimated the primary model after excluding the multivariate outliers identified in Section 2.3, using 2,100 bias-corrected bootstrap resamples.

## 3 Results

### 3.1 Descriptive statistics, reliability, and correlations

Table 1 reports descriptive statistics and internal consistency for all 12 wave-by-construct composites. Means were stable across waves for every construct, with no evidence of systematic drift. Skewness ranged from −0.59 to 0.24 and kurtosis from −0.73 to 0.62, well inside the range for which maximum likelihood estimation is robust. Reliability was good to excellent for attachment and prosocial behaviour at all three waves, acceptable to good for empathy, and acceptable for the six-item gratitude measure. Table 2 presents the full correlation matrix; all 66 correlations were positive and significant at *p* < 0.001, with the smallest at r = 0.17 and the largest at r = 0.70. As a criterion-related check on the project-specific prosocial inventory, scores showed the expected graded association with self-reported participation in school or community public-welfare activities. At Wave 1, mean prosocial scores were 4.384 for frequent participants (*n* = 101), 3.887 for occasional participants (*n* = 355), and 3.415 for those reporting no participation (*n* = 76), *F*(2, 529) = 41.31, *p* < 0.001, eta-squared = 0.135. The same ordering appeared at Wave 2 (4.457, 3.830, and 3.309; *F*(2, 501) = 56.74, *p* < 0.001, eta-squared = 0.185) and Wave 3 (4.406, 3.946, and 3.148; *F*(2, 531) = 62.66, *p* < 0.001, eta-squared = 0.191). Spearman correlations between the ordinal participation code and prosocial behaviour ranged from −0.395 to −0.421 across waves, all *p* < 0.001. Because both measures were self-reported, these findings strengthen within-sample criterion-related evidence but do not constitute independent external validation.

**Table 2:** Correlations among all study variables across three waves.

Variable	1	2	3	4	5	6	7	8	9	10	11	12
1. Parent–child attachment W1	1.00											
2. Parent–child attachment W2	0.678	1.00										
3. Parent–child attachment W3	0.667	0.643	1.00									
4. Empathy W1	0.217	0.171	0.221	1.00								
5. Empathy W2	0.294	0.340	0.317	0.655	1.00							
6. Empathy W3	0.299	0.326	0.414	0.642	0.642	1.00						
7. Gratitude W1	0.513	0.460	0.511	0.417	0.419	0.446	1.00					
8. Gratitude W2	0.432	0.532	0.449	0.319	0.484	0.437	0.579	1.00				
9. Gratitude W3	0.557	0.463	0.695	0.314	0.373	0.514	0.630	0.578	1.00			
10. Prosocial behaviour W1	0.509	0.407	0.393	0.295	0.245	0.312	0.494	0.359	0.431	1.00		
11. Prosocial behaviour W2	0.475	0.519	0.410	0.252	0.347	0.311	0.448	0.567	0.452	0.638	1.00	
12. Prosocial behaviour W3	0.497	0.369	0.539	0.263	0.261	0.396	0.420	0.355	0.581	0.640	0.606	1.00

W1, W2, and W3 denote the three waves. Pairwise n ranges from 483 to 536. All 66 correlations were significant at *p* < 0.001, so significance markers are omitted.

### 3.2 Longitudinal measurement invariance

Table 3 reports the invariance sequence for each construct. Configural models fitted well for attachment, gratitude and prosocial behaviour and acceptably for empathy. Metric invariance held without exception: the likelihood ratio test was non-significant for all four constructs, the smallest *p* value being 0.165, the largest absolute change in CFI was 0.002, and changes in RMSEA ranged from −0.008 to 0.003. Scalar constraints produced significant likelihood-ratio changes for empathy, Δχ^2^(4) = 11.74, *p* = 0.019, gratitude, Δχ^2^(4) = 14.09, *p* = 0.007, and prosocial behaviour, Δχ^2^(4) = 11.22, *p* = 0.024. Changes in CFI remained within the prespecified 0.010 criterion for all four constructs, but prosocial behaviour showed ΔRMSEA = 0.021 from the metric to the scalar model, exceeding the prespecified 0.015 criterion. Full scalar invariance therefore cannot be concluded for prosocial behaviour. Metric invariance was supported throughout, which is sufficient for the structural comparisons made here, but latent mean comparisons for prosocial behaviour are not treated as fully invariant.

**Table 3:** Longitudinal measurement invariance tests for each construct.

Construct	Model	χ^2^	df	CFI	TLI	RMSEA	SRMR	Δχ^2^	Δdf	p	ΔCFI	ΔRMSEA
Parent–child attachment	Configural	24.07	15	0.998	0.996	0.033	0.014					
Parent–child attachment	Metric	25.37	19	0.999	0.998	0.025	0.015	1.29	4	0.863	0.001	−0.008
Parent–child attachment	Scalar	33.78	23	0.998	0.997	0.029	0.016	8.41	4	0.078	−0.001	0.004
Empathy	Configural	56.36	15	0.985	0.963	0.071	0.032					
Empathy	Metric	61.14	19	0.984	0.970	0.063	0.034	4.78	4	0.310	−0.001	−0.008
Empathy	Scalar	72.88	23	0.982	0.971	0.063	0.037	11.74	4	0.019	−0.002	0.000
Gratitude	Configural	31.88	15	0.993	0.982	0.045	0.028					
Gratitude	Metric	38.37	19	0.991	0.984	0.043	0.034	6.49	4	0.165	−0.002	−0.002
Gratitude	Scalar	52.46	23	0.987	0.980	0.048	0.037	14.09	4	0.007	−0.004	0.005
Prosocial behaviour	Configural	14.84	15	1.000	1.000	0.000	0.011					
Prosocial behaviour	Metric	19.13	19	1.000	1.000	0.003	0.018	4.29	4	0.368	0.000	0.003
Prosocial behaviour	Scalar	30.35	23	0.999	0.998	0.024	0.019	11.22	4	0.024	−0.001	0.021

Δ values compare each model with the model immediately above it. Negative ΔRMSEA denotes improved fit under the added constraint. Prespecified criteria were |ΔCFI| ≤ 0.010 and ΔRMSEA ≤ 0.015. All models were estimated on the linked longitudinal sample (*N* = 557) using FIML.

### 3.3 Between-person and within-person structure

The traditional cross-lagged panel model fitted the data poorly, χ^2^(16) = 311.55, *p* < 0.001, CFI = 0.919, TLI = 0.667, RMSEA = 0.183 [0.166, 0.201], SRMR = 0.060. The random-intercept model fitted markedly better, χ^2^(6) = 28.00, *p* < 0.001, CFI = 0.994, TLI = 0.934, RMSEA = 0.082 [0.053, 0.113], SRMR = 0.025, and was favoured by 263.5 points on Akaike’s criterion and 220.4 points on the Bayesian criterion. Stable individual differences therefore account for substantial covariance that the traditional model forces into its lagged coefficients. As Figure 1 shows, random intercepts explained between 64.5 and 70.9% of total variance, so roughly two thirds of the variation in these constructs is stable between persons and only about one third is occasion-specific and available to the within-person structure.

**Figure 1:**
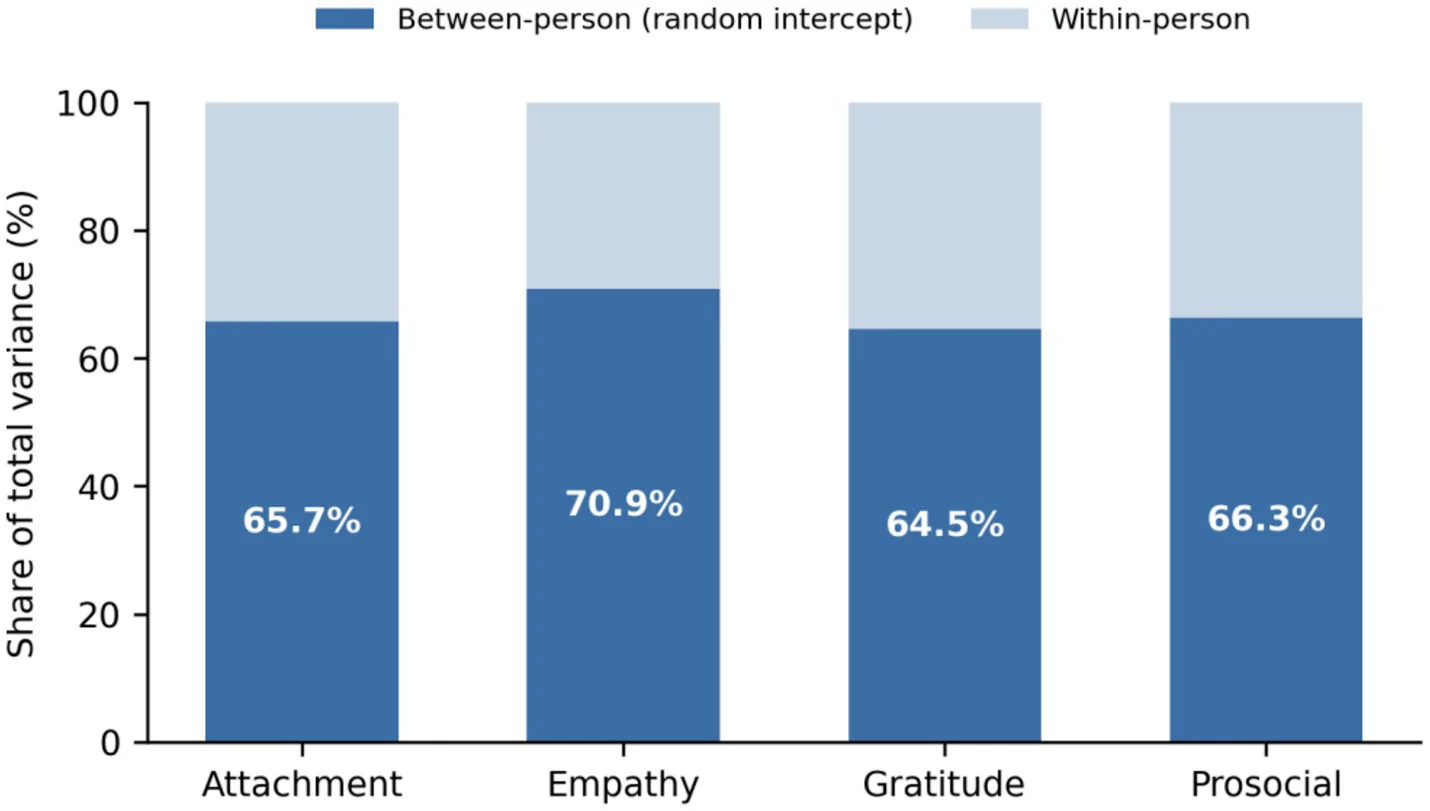
Proportion of variance attributable to stable between-person differences in the random-intercept cross-lagged panel model.

At the between-person level the four random intercepts were strongly and positively intercorrelated, as Table 4 shows. Adolescents with generally more secure attachment reported generally higher gratitude, r = 0.825, 95% CI [0.754, 0.896], and generally more prosocial behaviour, r = 0.676. Gratitude and prosocial behaviour were likewise closely tied at the trait level, r = 0.680. Empathy showed the weakest, though still substantial, links to the other three.

**Table 4:** Correlations among random intercepts in the random-intercept cross-lagged panel model.

Random intercept pair	r	SE	*p*	95% CI
Attachment with Empathy	0.405	0.051	< 0.001	[0.306, 0.504]
Attachment with Gratitude	0.825	0.036	< 0.001	[0.754, 0.896]
Attachment with Prosocial behaviour	0.676	0.037	< 0.001	[0.604, 0.748]
Empathy with Gratitude	0.598	0.041	< 0.001	[0.517, 0.679]
Empathy with Prosocial behaviour	0.444	0.049	< 0.001	[0.348, 0.540]
Gratitude with Prosocial behaviour	0.680	0.035	< 0.001	[0.613, 0.748]

Standardised estimates from the RI-CLPM, estimated on the linked longitudinal sample (*N* = 557) using FIML.

The within-person cross-lagged effects present a sharply different picture, reported in Table 5. Of the 32 estimated cross-lagged and autoregressive paths, only two reached conventional significance: within-person prosocial behaviour at Wave 1 negatively predicted within-person empathy at Wave 2, β = −0.232, *p* = 0.016, and within-person empathy at Wave 2 positively predicted within-person attachment at Wave 3, β = 0.256, *p* = 0.012. Neither lies on the hypothesised chain, and with 32 tests the expected number of false positives at the 0.05 level is 1.6, so both should be treated as provisional. The paths the hypotheses specify were not significant within persons: attachment to empathy, β = 0.076, *p* = 0.561; empathy to gratitude, β = 0.140, *p* = 0.160; gratitude to prosocial behaviour, β = −0.043, *p* = 0.694. This attenuation is consistent with H4, although H4 is not described as formally supported because no hypothesis-specific nested model comparison was conducted. The traditional model, by contrast, yielded the familiar chain, with Wave 1 attachment predicting Wave 2 empathy, β = 0.138, Wave 2 gratitude, β = 0.189, and Wave 2 prosocial behaviour, β = 0.174, all *p* < 0.001, and Wave 2 empathy predicting Wave 3 gratitude, β = 0.096, *p* = 0.015. The contrast between the two panels of Table 5 is the central empirical result of this study. The decomposition-based indirect checks led to the same conclusion. The prospective within-person indirect association from attachment W1 through empathy W2 to prosocial behaviour W3 was not distinguishable from zero, B = 0.0055, 95% CI [−0.0276, 0.0387], *p* = 0.744 (standardised product = 0.0047). The corresponding attachment W1 to gratitude W2 to prosocial behaviour W3 product was also not distinguishable from zero, B = 0.0089, 95% CI [−0.0407, 0.0585], *p* = 0.725 (standardised product = 0.0075). Thus, the mediation-like patterns that appear in the undecomposed latent model were not reproduced as prospective indirect products at the within-person level.

**Table 5:** Cross-lagged effects from the random-intercept and traditional cross-lagged panel models.

Path	β	SE	z	*p*	95% CI
Panel A. Within-person effects (RI-CLPM)					
Attachment W1 to Attachment W2	0.030	0.146	0.20	0.839	[−0.256, 0.316]
Empathy W1 to Attachment W2	−0.236	0.139	−1.70	0.089	[−0.509, 0.036]
Gratitude W1 to Attachment W2	−0.119	0.120	−0.99	0.322	[−0.354, 0.117]
Prosocial W1 to Attachment W2	−0.033	0.103	−0.33	0.745	[−0.234, 0.168]
Attachment W1 to Empathy W2	0.076	0.131	0.58	0.561	[−0.181, 0.333]
Empathy W1 to Empathy W2	−0.050	0.141	−0.35	0.724	[−0.325, 0.226]
Gratitude W1 to Empathy W2	0.036	0.117	0.31	0.757	[−0.194, 0.267]
Prosocial W1 to Empathy W2	−0.232	0.096	−2.42	0.016	[−0.420, −0.044]
Attachment W1 to Gratitude W2	−0.174	0.129	−1.35	0.178	[−0.426, 0.079]
Empathy W1 to Gratitude W2	−0.115	0.132	−0.87	0.385	[−0.374, 0.144]
Gratitude W1 to Gratitude W2	−0.012	0.124	−0.10	0.921	[−0.256, 0.231]
Prosocial W1 to Gratitude W2	−0.090	0.098	−0.92	0.358	[−0.283, 0.102]
Attachment W1 to Prosocial W2	−0.007	0.126	−0.06	0.956	[−0.253, 0.240]
Empathy W1 to Prosocial W2	−0.159	0.126	−1.26	0.208	[−0.407, 0.089]
Gratitude W1 to Prosocial W2	0.069	0.115	0.60	0.550	[−0.157, 0.294]
Prosocial W1 to Prosocial W2	−0.105	0.106	−0.99	0.322	[−0.312, 0.103]
Attachment W2 to Attachment W3	0.024	0.101	0.23	0.815	[−0.174, 0.221]
Empathy W2 to Attachment W3	0.256	0.102	2.52	0.012	[0.057, 0.456]
Gratitude W2 to Attachment W3	−0.112	0.093	−1.20	0.232	[−0.295, 0.071]
Prosocial W2 to Attachment W3	0.004	0.105	0.04	0.967	[−0.201, 0.209]
Attachment W2 to Empathy W3	0.113	0.099	1.14	0.254	[−0.081, 0.308]
Empathy W2 to Empathy W3	−0.055	0.104	−0.53	0.598	[−0.259, 0.149]
Gratitude W2 to Empathy W3	0.196	0.101	1.94	0.053	[−0.003, 0.394]
Prosocial W2 to Empathy W3	−0.097	0.103	−0.94	0.346	[−0.298, 0.105]
Attachment W2 to Gratitude W3	−0.154	0.085	−1.82	0.069	[−0.320, 0.012]
Empathy W2 to Gratitude W3	0.140	0.099	1.41	0.160	[−0.055, 0.335]
Gratitude W2 to Gratitude W3	−0.005	0.097	−0.05	0.962	[−0.194, 0.185]
Prosocial W2 to Gratitude W3	0.105	0.105	1.00	0.319	[−0.101, 0.310]
Attachment W2 to Prosocial W3	−0.148	0.103	−1.43	0.152	[−0.350, 0.054]
Empathy W2 to Prosocial W3	0.061	0.115	0.53	0.596	[−0.164, 0.286]
Gratitude W2 to Prosocial W3	−0.043	0.110	−0.39	0.694	[−0.260, 0.173]
Prosocial W2 to Prosocial W3	−0.078	0.124	−0.63	0.530	[−0.322, 0.166]
Panel B. Effects from the traditional CLPM					
Parent–child attachment W1 to Parent–child attachment W2	0.588	0.035	16.87	< 0.001	[0.520, 0.656]
Empathy W1 to Parent–child attachment W2	−0.041	0.036	−1.14	0.255	[−0.110, 0.029]
Gratitude W1 to Parent–child attachment W2	0.165	0.041	4.02	< 0.001	[0.085, 0.246]
Prosocial behaviour W1 to Parent–child attachment W2	0.048	0.039	1.23	0.220	[−0.029, 0.126]
Parent–child attachment W1 to Empathy W2	0.138	0.040	3.42	0.001	[0.059, 0.217]
Empathy W1 to Empathy W2	0.600	0.031	19.43	< 0.001	[0.539, 0.661]
Gratitude W1 to Empathy W2	0.130	0.042	3.06	0.002	[0.047, 0.213]
Prosocial behaviour W1 to Empathy W2	−0.083	0.040	−2.06	0.039	[−0.162, −0.004]
Parent–child attachment W1 to Gratitude W2	0.189	0.044	4.34	< 0.001	[0.104, 0.275]
Empathy W1 to Gratitude W2	0.081	0.040	2.06	0.040	[0.004, 0.159]
Gratitude W1 to Gratitude W2	0.441	0.043	10.16	< 0.001	[0.356, 0.526]
Prosocial behaviour W1 to Gratitude W2	0.031	0.044	0.70	0.481	[−0.055, 0.117]
Parent–child attachment W1 to Prosocial behaviour W2	0.174	0.041	4.27	< 0.001	[0.094, 0.254]
Empathy W1 to Prosocial behaviour W2	0.008	0.037	0.21	0.834	[−0.065, 0.080]
Gratitude W1 to Prosocial behaviour W2	0.120	0.043	2.76	0.006	[0.035, 0.205]
Prosocial behaviour W1 to Prosocial behaviour W2	0.494	0.038	13.09	< 0.001	[0.420, 0.568]
Parent–child attachment W2 to Parent–child attachment W3	0.545	0.037	14.60	< 0.001	[0.472, 0.618]
Empathy W2 to Parent–child attachment W3	0.066	0.038	1.77	0.077	[−0.007, 0.140]
Gratitude W2 to Parent–child attachment W3	0.104	0.045	2.31	0.021	[0.016, 0.193]
Prosocial behaviour W2 to Parent–child attachment W3	0.052	0.042	1.23	0.218	[−0.031, 0.135]
Parent–child attachment W2 to Empathy W3	0.055	0.042	1.33	0.185	[−0.026, 0.137]
Empathy W2 to Empathy W3	0.559	0.033	16.81	< 0.001	[0.494, 0.624]
Gratitude W2 to Empathy W3	0.129	0.046	2.83	0.005	[0.040, 0.218]
Prosocial behaviour W2 to Empathy W3	0.013	0.043	0.30	0.766	[−0.071, 0.096]
Parent–child attachment W2 to Gratitude W3	0.178	0.043	4.15	< 0.001	[0.094, 0.261]
Empathy W2 to Gratitude W3	0.096	0.039	2.44	0.015	[0.019, 0.172]
Gratitude W2 to Gratitude W3	0.376	0.046	8.22	< 0.001	[0.286, 0.465]
Prosocial behaviour W2 to Gratitude W3	0.123	0.044	2.80	0.005	[0.037, 0.209]
Parent–child attachment W2 to Prosocial behaviour W3	0.079	0.044	1.82	0.069	[−0.006, 0.165]
Empathy W2 to Prosocial behaviour W3	0.061	0.040	1.52	0.129	[−0.018, 0.139]
Gratitude W2 to Prosocial behaviour W3	−0.031	0.048	−0.64	0.520	[−0.125, 0.063]
Prosocial behaviour W2 to Prosocial behaviour W3	0.566	0.040	14.01	< 0.001	[0.487, 0.645]

β = standardised coefficient. Panel A estimates are among within-person deviations; Panel B estimates are among observed scores. Both models were estimated on the linked longitudinal sample (*N* = 557) using FIML.

### 3.4 Latent sequential indirect-effect model

The latent model fitted well, χ^2^(199) = 430.75, *p* < 0.001, CFI = 0.974, TLI = 0.967, RMSEA = 0.046 [0.040, 0.052], SRMR = 0.046, and accounted for 56.3% of the variance in Wave 2 empathy, 58.9% in Wave 3 gratitude and 54.9% in Wave 3 prosocial behaviour. Figure 2 shows the specification with its estimated coefficients, and Table 6 reports all structural paths. Consistent with H1, Wave 1 attachment was positively associated with Wave 2 empathy after adjustment for Wave 1 empathy, B = 0.100, bias-corrected 95% CI [0.032, 0.163], β = 0.141. Consistent with H2, Wave 2 empathy was positively associated with Wave 3 gratitude after adjustment for Wave 1 gratitude and attachment, B = 0.171, 95% CI [0.015, 0.329], β = 0.117. Attachment was also associated with Wave 3 gratitude, B = 0.282, 95% CI [0.147, 0.398], β = 0.271, and gratitude with Wave 3 prosocial behaviour, B = 0.376, 95% CI [0.241, 0.545], β = 0.365. Empathy was not independently associated with prosocial behaviour, B = −0.020, 95% CI [−0.151, 0.107], and the direct attachment coefficient was not distinguishable from zero, B = 0.056, 95% CI [−0.061, 0.176].

**Figure 2:**
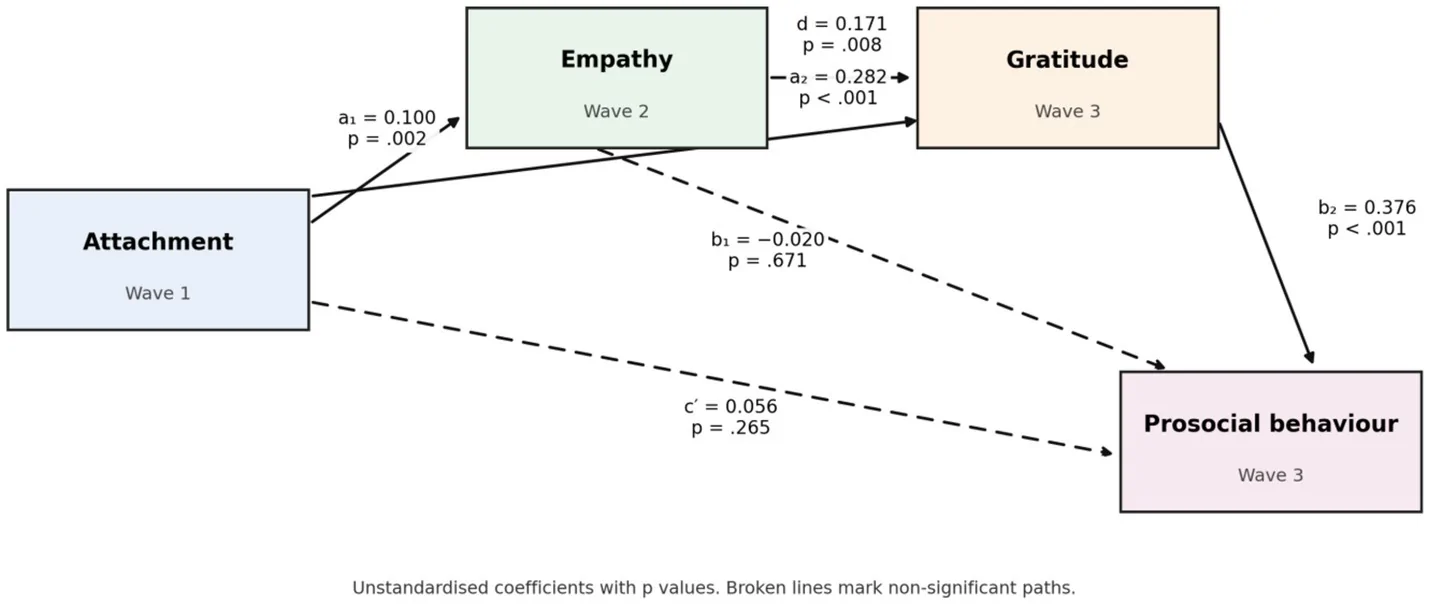
Three-wave latent sequential indirect-effect model with estimated coefficients. Coefficients are unstandardised estimates, each shown with its *p* value. Wave 1 autoregressive controls for each intervening construct and the outcome, together with gender and age band, were estimated but are omitted from the figure for readability. Broken lines mark non-significant paths.

**Table 6:** Structural paths in the latent sequential indirect-effect model.

Path	Label	B	Boot SE	β	Bias-corrected 95% CI	CI excludes 0
Attachment W1 to Empathy W2	a1	0.100	0.033	0.141	[0.032, 0.163]	Yes
Empathy W1 to Empathy W2	arE	0.757	0.063	0.689	[0.641, 0.883]	Yes
Empathy W2 to Gratitude W3	dm	0.171	0.079	0.117	[0.015, 0.329]	Yes
Attachment W1 to Gratitude W3	a2	0.282	0.064	0.271	[0.147, 0.398]	Yes
Gratitude W1 to Gratitude W3	arG	0.566	0.093	0.520	[0.388, 0.753]	Yes
Empathy W2 to Prosocial W3	b1	−0.020	0.066	−0.013	[−0.151, 0.107]	No
Gratitude W3 to Prosocial W3	b2	0.376	0.076	0.365	[0.241, 0.545]	Yes
Attachment W1 to Prosocial W3 (direct)	cp	0.056	0.060	0.053	[−0.061, 0.176]	No
Prosocial W1 to Prosocial W3	arP	0.491	0.058	0.457	[0.377, 0.605]	Yes

B = unstandardised estimate. Boot SE = bootstrap standard deviation across 5,000 resamples. β = standardised estimate. Gender and age band were included as covariates. Estimated on *N* = 557 linked adolescents using FIML.

Table 7 decomposes the model-implied association into direct and indirect components, and Figure 3 displays all effects with their intervals. The largest indirect association operated through gratitude without empathy, B = 0.1058, 95% CI [0.0485, 0.1935]. The empathy-only route was not distinguishable from zero, B = −0.0020, 95% CI [−0.0177, 0.0109]. The sequential product through empathy and gratitude was statistically detectable in the primary model, B = 0.0064, 95% CI [0.0010, 0.0202], but very small, representing approximately 5.8% of the total indirect association of B = 0.1102, 95% CI [0.0526, 0.1963]. The total association was B = 0.1666, 95% CI [0.0602, 0.2685]. Inference on an indirect effect concerns the product of coefficients and does not require every constituent coefficient to be individually significant. The direct coefficient was not distinguishable from zero while the total indirect association was detectable, which does not by itself establish full mediation but indicates that most of the model-implied indirect association ran through gratitude. Figure 4 shows the bootstrap distribution of the sequential product.

**Table 7:** Direct, indirect and total effects with bias-corrected bootstrap confidence intervals.

Effect	B	Boot SE	β	Bias-corrected 95% CI	Percentile 95% CI	Significant
Direct effect (c′)	0.0564	0.0604	0.053	[−0.0612, 0.1763]	[−0.0634, 0.1745]	No
Indirect via empathy only (a1 × b1)	−0.0020	0.0070	−0.002	[−0.0177, 0.0109]	[−0.0165, 0.0120]	No
Indirect via gratitude only (a2 × b2)	0.1058	0.0363	0.099	[0.0485, 0.1935]	[0.0462, 0.1873]	Yes
Sequential indirect (a1 × d × b2)	0.0064	0.0045	0.006	[0.0010, 0.0202]	[0.0002, 0.0177]	Yes
Total indirect	0.1102	0.0364	0.103	[0.0526, 0.1963]	[0.0502, 0.1906]	Yes
Total effect	0.1666	0.0528	0.156	[0.0602, 0.2685]	[0.0631, 0.2696]	Yes

Based on 5,000 bootstrap resamples, all of which converged. Bias-corrected and percentile intervals are both reported so that the size of the bias adjustment is visible. Analytic sample *N* = 557.

**Figure 3:**
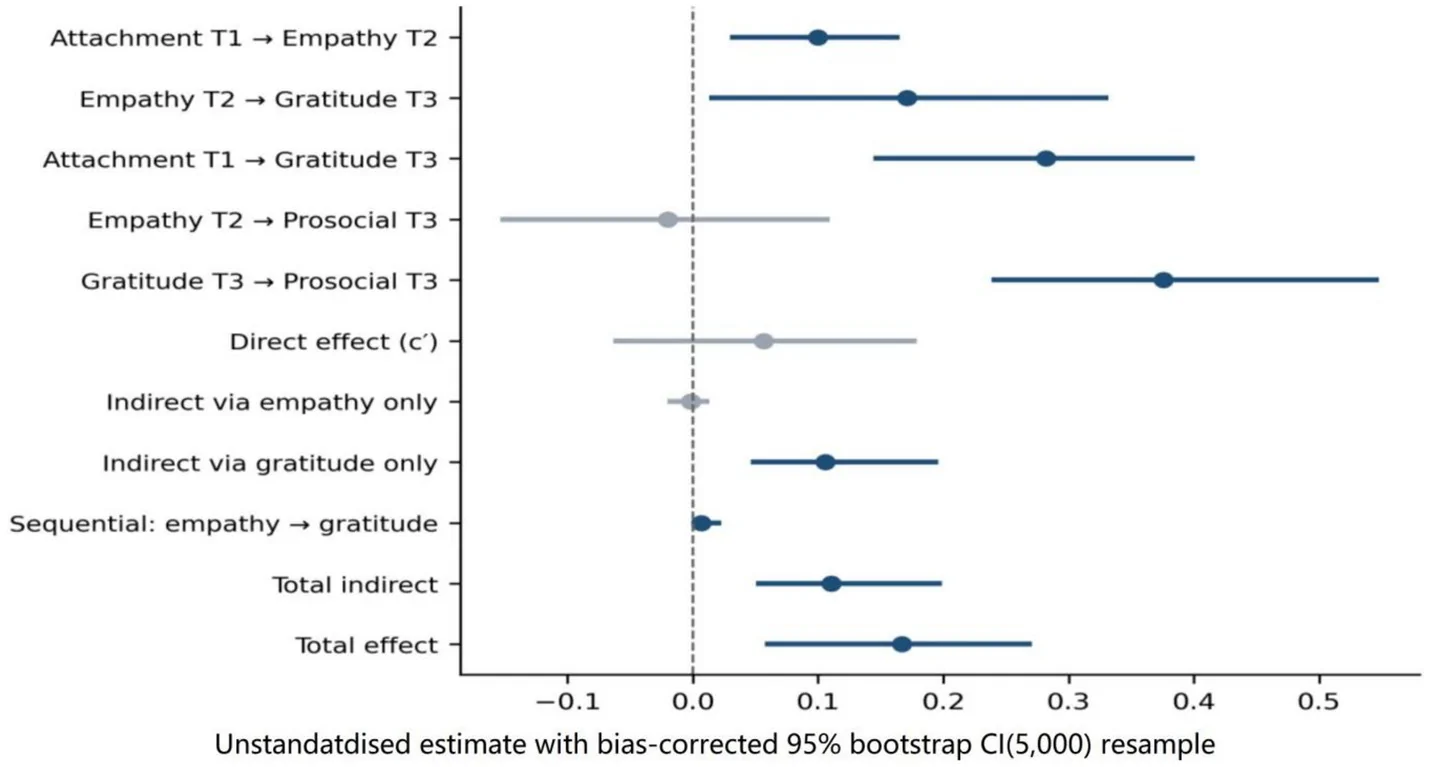
Direct, indirect and total effects with bias-corrected bootstrap intervals.

**Figure 4:**
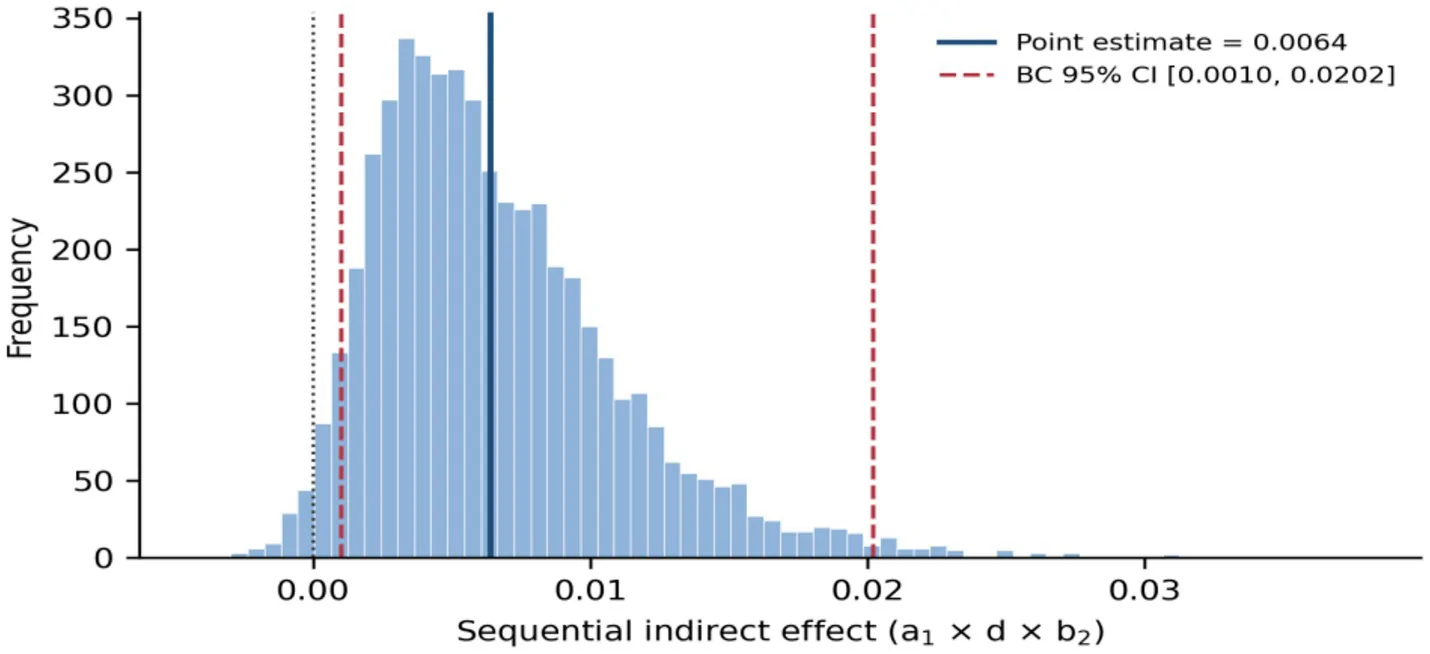
Bootstrap distribution of the sequential indirect association across 5,000 resamples.

#### 3.4.1 Empathy-facet sensitivity analysis

The facet analysis confirmed that the total empathy score should not be interpreted as a homogeneous mechanism. Wave 1 attachment was associated with both affective empathy at Wave 2 (B = 0.125, *p* < 0.001) and cognitive empathy at Wave 2 (B = 0.138, *p* = 0.001). Affective empathy was subsequently associated with Wave 3 gratitude (B = 0.114, *p* = 0.027), whereas cognitive empathy was not (B = 0.059, *p* = 0.179). Neither facet showed an independent association with Wave 3 prosocial behaviour once gratitude was included. The broad empathy association therefore reflected contributions from both facets at the attachment-to-empathy step, but the later empathy-to-gratitude association was concentrated in affective empathy in this sensitivity analysis.

### 3.5 Robustness

Moving the second intervening construct one wave earlier changed the result substantially. Fit remained good in the alternative model, CFI = 0.976, TLI = 0.970, RMSEA = 0.043, SRMR = 0.044. Wave 1 attachment remained associated with Wave 2 empathy, β = 0.146, and Wave 2 empathy with Wave 2 gratitude, β = 0.322, *p* < 0.001, although those two constructs were now concurrent rather than one wave apart. The path from gratitude to prosocial behaviour fell to non-significance, B = 0.061, 95% CI [−0.040, 0.162], β = 0.062, *p* = 0.234, while the direct effect of attachment became significant, B = 0.209, β = 0.195, *p* < 0.001. The total indirect association remained detectable, B = 0.0231, 95% CI [0.0016, 0.0445], but was roughly one fifth the size of the corresponding primary estimate, and no individual indirect path was significant. The clearly lagged attachment-to-gratitude-to-prosocial association was not distinguishable from zero, B = 0.0107, 95% CI [−0.0080, 0.0294], so H3a was not supported.

The second robustness analysis excluded the 26 multivariate outliers and refitted the primary model on the remaining 531 adolescents with 2,100 bias-corrected bootstrap resamples. Fit was comparable, CFI = 0.975, RMSEA = 0.045, SRMR = 0.046. Three of the four significant structural paths held: attachment to empathy, B = 0.086, 95% CI [0.028, 0.142]; attachment to gratitude, B = 0.224, 95% CI [0.094, 0.354]; and gratitude to prosocial behaviour, B = 0.322, 95% CI [0.212, 0.472]. The indirect route through gratitude alone also held, B = 0.0722, 95% CI [0.0297, 0.1415], as did the total indirect association, B = 0.0751, 95% CI [0.0316, 0.1439]. The empathy-to-gratitude coefficient, however, became non-significant, B = 0.162, 95% CI [−0.020, 0.333], while the interval for the sequential product remained narrowly above zero, B = 0.0045, 95% CI [0.0002, 0.0149]. These results are not statistically contradictory, but the sequential estimate was extremely small, its lower bound was very close to zero, and the same product was not detectable under the alternative temporal specification. Table 8 places the three specifications side by side.

**Table 8:** Comparison of the primary, alternative and outlier-excluded specifications.

Quantity	Primary model	Alternative model	Outliers excluded
Sample	All 557	All 557	531 (outliers excluded)
Gratitude measured at	Wave 3	Wave 2	Wave 3
Interval method	BC bootstrap, 5,000	Maximum likelihood	BC bootstrap, 2,100
CFI	0.974	0.976	0.975
RMSEA	0.046	0.043	0.045
SRMR	0.046	0.044	0.046
Attachment to empathy (a1)	0.100 [0.032, 0.163]	0.104 [0.047, 0.161]	0.086 [0.028, 0.142]
Empathy to gratitude (d)	0.171 [0.015, 0.329]	0.491 [0.336, 0.647]	0.162 [−0.020, 0.333]
Gratitude to prosocial (b2)	0.376 [0.241, 0.545]	0.061 [−0.040, 0.162]	0.322 [0.212, 0.472]
Direct effect (c′)	0.056 [−0.061, 0.176]	0.209 [0.114, 0.304]	0.089 [−0.022, 0.202]
Indirect via gratitude	0.1058 [0.0485, 0.1935]	0.0107 [−0.0080, 0.0294]	0.0722 [0.0297, 0.1415]
Sequential indirect	0.0064 [0.0010, 0.0202]	0.0031 [−0.0024, 0.0086]	0.0045 [0.0002, 0.0149]
Total indirect	0.1102 [0.0526, 0.1963]	0.0231 [0.0016, 0.0445]	0.0751 [0.0316, 0.1439]

Values are unstandardised estimates with 95% intervals. BC = bias-corrected. The alternative specification was fitted with maximum likelihood intervals and was not bootstrapped, so its intervals are not directly comparable with the bootstrap intervals in the other two columns.

## 4 Discussion

This study examined whether longitudinal associations among parent–child attachment, empathy, gratitude and prosocial behaviour are consistent with a proposed sequential account, and whether the corresponding associations are evident within persons. The results do not establish a complete attachment to empathy to gratitude to prosocial behaviour developmental mechanism. In the conventional latent model the constituent associations and the sequential product were statistically detectable in the primary specification, but the product was very small and depended on a specification in which gratitude and prosocial behaviour were measured concurrently. The alternative temporal specification did not reproduce it, and the empathy-to-gratitude component became non-significant when influential cases were excluded. More importantly, the RI-CLPM provided no evidence for the hypothesised within-person cross-lagged chain, and the two prospective within-person indirect products that are identifiable with three waves were also not distinguishable from zero. The most consistent finding is therefore a pattern of stable longitudinal associations, particularly those involving attachment and gratitude, rather than a clearly identified within-person developmental process.

The pattern of coefficients is nonetheless informative about which association was most consistent in the undecomposed latent model. Gratitude showed the strongest proximal relation to prosocial behaviour, and the indirect route through gratitude alone accounted for most of the model-implied indirect association, whereas empathy had no independent direct route to behaviour once gratitude was included. This pattern is descriptively compatible with the find-remind-and-bind account (Algoe, 2012), but it does not establish that gratitude is the mechanism through which empathy influences prosocial behaviour. Empathy may register another person’s state, whereas gratitude adds an appraisal of benefaction; however, the present observational data cannot establish that appraisal sequence. The broaden-and-build framework offers a plausible accumulation account (Fredrickson, 2001), but it remains a theoretical interpretation rather than a demonstrated mechanism here. The absence of an unmediated empathy route also converges with work showing that empathy relates unevenly to behaviour depending on which component is measured (Håkansson Eklund and Summer Meranius, 2021; Palumbo and Latzman, 2021). The ordering should not be pressed too hard, because the empathy-to-gratitude link was the weakest coefficient in the model, the facet analysis suggested that the association was concentrated in affective rather than cognitive empathy, and the coefficient lost significance when influential cases were removed.

The attachment-to-gratitude association was comparatively strong, roughly three times the magnitude of the attachment-to-empathy path, with a between-person correlation between the corresponding random intercepts of r = 0.825. This indicates substantial trait-level overlap but not empirical identity, and item-level heterotrait-monotrait values remained below the conservative 0.85 criterion at all three waves, with a maximum of 0.812. The strength of the association nevertheless warrants caution because both constructs were assessed by adolescent self-report within the same sessions. The direction is consistent with prospective attachment effects on gratitude (Li et al., 2023) and with gratitude placed downstream of attachment in predicting life satisfaction (Lin, 2020). It also raises a measurement question this design cannot settle, namely how far a gratitude questionnaire administered to adolescents captures something distinct from a generalised positive appraisal of one’s relational world, particularly since a six-item global measure may blend cognitive and affective elements that behave differently (Zhang L. et al., 2022).

Three explanations for the null within-person effects are worth weighing. The first is substantive: these constructs may simply be slow-moving dispositions during this period, so that an adolescent briefly above their own usual level of gratitude does not become measurably more prosocial a wave later. The variance decomposition supports that reading, since roughly two thirds of the variance in each construct was stable, leaving a modest within-person signal. The second is methodological: three waves is the identification minimum for a random-intercept model, the within-person paths are estimated with wide intervals, several of which remain compatible with effects of practical interest, and the near-zero within-person autoregressive coefficients indicate that occasion-specific deviations did not persist from one wave to the next. The third concerns lag length, since cross-lagged coefficients are lag-dependent and processes operating over weeks would be missed by a panel spaced more widely. A previous study using both models on gratitude and prosocial behaviour in Chinese adolescents found the same between-person and within-person divergence (Yang et al., 2021), so two independent samples now show the pattern, which makes a purely idiosyncratic explanation less plausible. Whichever explanation holds, the practical lesson is the same: the traditional model in this dataset reproduced exactly the tidy chain the literature reports, and did so while fitting the data poorly and while being outperformed on both information criteria, which echoes accumulated warnings about cross-lagged inference (Lucas, 2023; Orth et al., 2021) and supports interpreting within-person estimates alongside rather than instead of other evidence (Sorjonen and Melin, 2022).

The robustness analysis carries a caution for this literature more broadly. When gratitude and prosocial behaviour were measured on the same occasion the path between them was large and highly significant; when gratitude was moved one wave earlier so that it genuinely preceded the outcome, that path fell to non-significance and the direct effect of attachment reasserted itself. Part of the concurrent association may therefore reflect shared method and shared occasion rather than transmission over time, although the present design cannot quantify that contribution. Models that place an intervening construct and the outcome at the same wave may overstate the mediated portion, so reporting both temporal placements is a useful sensitivity check rather than an optional extra.

Two practical implications follow, both stated as hypotheses for future experimental work rather than as established intervention mechanisms. For parenting programmes, the results suggest that improving communication or warmth should not be assumed to produce helping behaviour on its own. Gratitude showed the most consistent statistical association with prosocial behaviour in the models examined here, which makes it a plausible target, and expressed gratitude is known to produce measurable wellbeing benefits (Kirca et al., 2023), with prosocial behaviour a reasonable additional outcome to measure. Expectations should stay modest, since a large registered replication found the wellbeing return of spending on others to be smaller and more conditional than earlier work implied (Aknin et al., 2020), and the returns of helping appear to depend on whether the motivation behind it is autonomous (Zhang et al., 2024). For school-based social–emotional learning, the absence of an independent empathy-to-prosocial path in the primary latent model is informative but should not be interpreted as showing that empathy-focused programmes are ineffective. In this sample, empathy had no independent direct association with prosocial behaviour once gratitude was included, and the facet sensitivity analysis indicated that only affective empathy was associated with later gratitude. These findings motivate a testable comparison between empathy-focused programmes and programmes that additionally target grateful appraisal. The present observational results do not establish that adding gratitude training would causally increase prosocial behaviour.

Beyond replication, two design directions would address the interpretive limits identified here. An intensive longitudinal design, with daily or weekly assessments nested within a longer panel, would allow the gratitude-to-prosociality association to be estimated at the timescale on which the emotion plausibly operates and would show whether the null within-person findings reflect a genuinely trait-like process or a lag mismatch. A randomised gratitude intervention in a school setting, with parent-reported and peer-nominated prosocial behaviour as outcomes alongside self-report and with an empathy-only comparison arm, would break the common-method dependence and convert the associations reported here into evidence about manipulability. Broadening the outcome to include online helping would also be worthwhile, given evidence that adolescents’ perceptions of digital settings shape whether they help there (Pastor et al., 2025).

### 4.1 Limitations

Several limitations qualify these conclusions. First, all measures were adolescent self-reports collected in the same session with the same response format, so shared method variance may inflate associations and is a plausible partial explanation for the high between-person correlation between attachment and gratitude. The first-unrotated-factor diagnostic is only descriptive and cannot rule out common-method bias; no marker variable, alternate informant, or alternate response format was available. Stable response style may therefore contribute to the random-intercept correlations and the undecomposed latent indirect associations, while time-varying method effects can remain even after the RI-CLPM separates stable between-person variance. Prosocial behaviour is particularly vulnerable to socially desirable responding, and the observed means, all near four on a five-point scale, are consistent with some positivity bias. Second, the prosocial behaviour inventory has no identified published source scale. Its one-factor structure, high internal consistency, positive associations with the other focal constructs, and graded association with reported participation in public-welfare activities provide supportive within-sample evidence, but the participation criterion was also self-reported and does not substitute for validation against an established instrument or independent behavioural criterion. The attachment instrument also carries the wording anomaly described in Section 2.2.1. Third, the archived materials do not permit reconstruction of the original item-selection and Chinese translation procedures for the attachment measure, so it is not presented as a formally validated short form, and the incomplete documentation limits measurement reproducibility.

Fourth, the design has three waves, which is the identification minimum for the random-intercept model and leaves the within-person estimates imprecise; it also forced the second intervening construct and the outcome to be measured concurrently in the primary specification. The calendar spacing between waves is not documented in the archived materials, so the lag over which these processes were observed cannot be stated. This is a central interpretive limitation because cross-lagged magnitudes are lag-dependent and the null within-person results could partly reflect a mismatch between the measurement interval and the timescale on which these processes operate. Fifth, the exploratory sequential association specified in H3b was small and sensitive to influential observations, since excluding 26 multivariate outliers rendered the empathy-to-gratitude coefficient non-significant even though the bootstrap interval for the product remained narrowly above zero; together with the failure to replicate under the alternative temporal specification, this warrants cautious interpretation and independent replication. Sixth, although FIML is preferable to listwise deletion under a missing-at-random mechanism, Little’s test rejected missing completely at random and the missing-at-random assumption cannot be demonstrated from observed data, so results may remain sensitive to unobserved causes of missingness. Seventh, the sample comes from a single Chinese junior secondary setting concentrated in one grade cohort, and filial obligation and family interdependence are salient in this cultural context, so generalisation should be cautious. Finally, these data are observational: the random-intercept decomposition removes stable between-person confounding but does not address time-varying confounding, and separating within-person from between-person variance clarifies which question a coefficient answers without licensing a causal claim (Rohrer and Murayama, 2023).

## 5 Conclusion

Across three waves of data from 557 Chinese adolescents, parent–child attachment, empathy, gratitude and prosocial behaviour showed meaningful longitudinal associations, but the results do not establish a complete and temporally ordered attachment to empathy to gratitude to prosocial behaviour mechanism. In the conventional latent model the sequential indirect association was statistically detectable in the primary specification, B = 0.0064, 95% CI [0.0010, 0.0202], but represented only about 5.8% of the total indirect association. Gratitude and prosocial behaviour were measured concurrently in that model, the sequential product was not reproduced when gratitude was placed at the preceding wave, and the empathy-to-gratitude component became non-significant after influential cases were excluded. The random-intercept model likewise gave no support for the hypothesised within-person cross-lagged chain, with random intercepts accounting for 64.5 to 70.9% of total variance, and the two prospective within-person indirect products identifiable across three waves were also not distinguishable from zero. The evidence is therefore strongest for stable associations among adolescents, particularly those involving attachment and gratitude, rather than for a clearly identified within-person developmental sequence. A four-wave or longer design placing attachment, empathy, gratitude and prosocial behaviour on distinct occasions, ideally supplemented by intensive longitudinal or experimental data, is needed to test the proposed temporal mechanism directly.

The de-identified participant-level dataset cannot be posted publicly because it was collected from minors and remains subject to the conditions of the original ethical approval described in Section 2.1. Researchers may request controlled access from the corresponding author; requests will be reviewed for scientific purpose, institutional approval and compliance with an appropriate data-use agreement. The complete analysis code, comprising the Python data-preparation scripts and the lavaan model syntax for every model reported here, is provided as Supplementary Table S4, together with software-version information and bootstrap settings. A variable dictionary and key model outputs will be supplied with approved data requests.

## Data Availability

The raw data supporting the conclusions of this article will be made available by the authors, without undue reservation.
