# Supplementary material for: Longitudinal associations between parent–child attachment and prosocial behaviour: the roles of empathy and gratitude
Source: Front Psychol. 2026 Oct 21;17:1970907. doi: 10.3389/fpsyg.2026.1970907 (PMC13730822; doi:10.3389/fpsyg.2026.1970907)
Supplement: Supplementary file 1 [file Supplementary_file_1.docx]

**Supplementary Material**

**Longitudinal Associations Between Parent-Child Attachment and Prosocial Behavior: The Roles of Empathy and Gratitude**

This file contains Supplementary Tables S1 to S4 cited in the main text. The complete runnable analysis code of Supplementary Table S4 is additionally supplied as a separate archive, Analysis_Code.zip.

**Supplementary Table S1.** Complete administered item set of the 15-item prosocial-behaviour inventory (C1-C15)

| **Item** | **Item content** |
| --- | --- |
| C1 | I give up my seat to people who need it, such as the elderly, the frail, the sick, the disabled and pregnant women |
| C2 | When a classmate is unwell, I take them to the school clinic |
| C3 | I help classmates catch up on lessons or teach them to play ball games |
| C4 | I am glad to donate money or goods to disaster-stricken areas |
| C5 | I like taking part in public-service activities organised inside or outside school |
| C6 | I am willing to do things for my class as a collective |
| C7 | I appreciate my parents’ hard work and take on household chores within my ability |
| C8 | When on cleaning duty, I am willing to spend extra time cleaning the classroom |
| C9 | I greet teachers and elders when I meet them |
| C10 | I invite bystanders to join in our games |
| C11 | I greet new classmates and make friends with them |
| C12 | After a minor disagreement with a friend, I apologise first |
| C13 | I am willing to correct my own shortcomings |
| C14 | I keep other people’s secrets |
| C15 | I often praise other people’s strengths |

*Note:* The inventory was administered in Chinese; the wording shown here is an English translation prepared for this report and was not the form presented to participants. Items are listed in the order administered. All 15 items are positively worded and were rated on a five-point scale, and none is reverse-scored. The original Chinese item wording is available from the corresponding author under the controlled-access procedure described in the Availability of Data and Materials statement.

**Supplementary Table S2.** Parcel construction used in the longitudinal latent models

| **Construct** | **Item pool** | **Parcels** | **Items per parcel** | **Balancing constraint applied** |
| --- | --- | --- | --- | --- |
| Parent-child attachment | D1-D20 (20 items) | 3 | 7, 7, 6 | Each parcel draws from both the mother-referenced items (D1, D3, D5, D7, D9, D11, D13, D15, D17, D19) and the father-referenced items (D2, D4, D6, D8, D10, D12, D14, D16, D18, D20), so that no parcel is specific to one parent. |
| Empathy | A1-A20 (20 items) | 3 | 7, 7, 6 | Each parcel draws from both the affective subscale (A1, A2, A4, A5, A7, A8, A11, A13, A15, A17, A18) and the cognitive subscale (A3, A6, A9, A10, A12, A14, A16, A19, A20), so that no parcel represents a single facet. |
| Gratitude | B1-B6 (6 items) | 3 | 2, 2, 2 | The six items form three equally sized parcels; the two negatively keyed items (B3, B6) are not placed in the same parcel. |
| Prosocial behaviour | C1-C15 (15 items) | 3 | 5, 5, 5 | The fifteen items form three equally sized parcels spanning the school, family and community content shown in Supplementary Table S1. |

*Note:* Parcels are unweighted means of their constituent items, computed under the same 60% item-completion rule applied to the full scale scores. The allocation was fixed on the Wave 1 data and applied unchanged to Waves 2 and 3, which is the condition required for the longitudinal invariance tests reported in Section 3.2.

Within each facet, items were ordered by their item-total correlation at Wave 1 and dealt into the three parcels in serpentine order, so that every parcel draws proportionally from every facet and the parcels are balanced on loading strength. The resulting item-level assignment, which is reproduced exactly by script 01 of Supplementary Table S4, is listed below.

| **Construct** | **Parcel** | **Items** | **Item assignment** |
| --- | --- | --- | --- |
| Parent-child attachment | Parcel 1 | 7 | D1, D6, D7, D16, D18, D19, D20 |
|  | Parcel 2 | 7 | D2, D9, D10, D12, D14, D15, D17 |
|  | Parcel 3 | 6 | D3, D4, D5, D8, D11, D13 |
| Empathy | Parcel 1 | 7 | A4, A5, A9, A10, A16, A17, A20 |
|  | Parcel 2 | 7 | A1, A3, A8, A14, A15, A18, A19 |
|  | Parcel 3 | 6 | A2, A6, A7, A11, A12, A13 |
| Gratitude | Parcel 1 | 2 | B1, B6 |
|  | Parcel 2 | 2 | B2, B3 |
|  | Parcel 3 | 2 | B4, B5 |
| Prosocial behaviour | Parcel 1 | 5 | C1, C5, C9, C12, C13 |
|  | Parcel 2 | 5 | C4, C6, C7, C10, C15 |
|  | Parcel 3 | 5 | C2, C3, C8, C11, C14 |

*Note:* Item prefixes denote the instrument: A = Basic Empathy Scale, B = Gratitude Questionnaire, C = prosocial-behaviour inventory, D = parent-child attachment measure.

**Supplementary Table S3.** Attrition analysis comparing retained and non-retained participants on Wave 1 scores

| **Wave 1 variable** | **Retained M (SD)** | **Non-retained M (SD)** | **t** | **df** | **p** | **Cohen's d** |
| --- | --- | --- | --- | --- | --- | --- |
| Parent-child attachment | 3.820 (0.743) | 3.801 (0.797) | 0.173 | 68.2 | 0.863 | 0.025 |
| Empathy | 3.472 (0.522) | 3.423 (0.528) | 0.664 | 69.8 | 0.509 | 0.094 |
| Gratitude | 3.684 (0.767) | 3.747 (0.763) | -0.585 | 68.8 | 0.560 | -0.083 |
| Prosocial behaviour | 3.913 (0.753) | 3.923 (0.823) | -0.093 | 67.7 | 0.926 | -0.013 |

*Note:* Retained n = 475 participants with valid scores on all 12 wave-by-construct composites; non-retained n = 57 (n = 56 for gratitude) Wave 1 records with a valid baseline score for the variable compared. Welch t tests with unequal variances. No comparison approached significance and all effect sizes were negligible.

**Supplementary Table S4.** Complete analysis code, software versions and file manifest

Every result reported in the manuscript was produced by the scripts listed below. They are reproduced in full in this file and are also supplied as a separate runnable archive (Analysis_Code.zip). Running scripts 01 to 07 in order, in a single working directory containing the source workbook, regenerates every table, figure and reported statistic.

**S4.1 Software versions**

| **Component** | **Version** | **Used for** |
| --- | --- | --- |
| R | 4.3.3 | All structural equation models |
| R package lavaan | 0.6-17 | Measurement invariance, RI-CLPM, latent indirect-effect models, bootstrap |
| Python | 3.12.3 | Data preparation, reliability, diagnostics, figures |
| pandas | 3.0.2 | Data handling |
| numpy | 2.4.4 | Numerical routines |
| scipy | 1.17.1 | Distributions and significance tests |
| matplotlib | 3.10.8 | Figures at 300 dpi |

*Note:* Estimation used full-information maximum likelihood throughout. The random seed was fixed at 20260804 for every model and every bootstrap routine.

**S4.2 File manifest**

| **Script** | **Purpose** | **Produces** |
| --- | --- | --- |
| 01_prepare_data.py | Reads the three-wave workbook, cleans and reverse-scores items, links waves on the participant identifier, forms scale scores under the 60% completion rule, and builds the three domain-representative parcels per construct | analytic.csv, wide_all.csv |
| 02_descriptives_reliability.py | Cronbach alpha, McDonald omega, descriptive statistics, correlation matrix, Little MCAR test, attrition comparison, Mahalanobis outlier screening | Table 1, Table 2, Table S3, outliers.csv |
| 03_invariance.R | Configural, metric and scalar longitudinal invariance for each construct | Table 3 |
| 04_riclpm.R | Random-intercept cross-lagged panel model and the traditional cross-lagged panel model | Table 4, Table 5, Fig. 1 |
| 05_indirect_effect.R | Latent sequential indirect-effect model with autoregressive controls and 5,000 bias-corrected bootstrap resamples | Table 6, Table 7, Figs. 2-4 |
| 06_robustness.R | Alternative temporal placement of gratitude, and the outlier-excluded refit with 2,100 bias-corrected bootstrap resamples | Table 8 |
| 07_figures.py | Generates the four figures at 300 dpi | Figs. 1-4 |

*Note:* The source workbook is not distributed with this code. It is available from the corresponding author under the controlled-access procedure described in the Availability of Data and Materials statement.

**S4.3 Full code listings**

```
01_prepare_data.py
"""
01_prepare_data.py
Reads the three-wave workbook, cleans it, reverse-scores, links waves,
builds scale scores and the three domain-representative parcels per construct,
and writes the analysis file used by every R script.

Input : 总表.xlsx  (worksheets 第1波 / 第2波 / 第3波, one per wave)
Output: analytic.csv  (N = 557 adolescents linked across all three waves)
        wide_all.csv  (all records, used for wave-level descriptives)
"""
import pandas as pd, numpy as np

SRC    = "总表.xlsx"
SHEETS = {"T1": "第1波", "T2": "第2波", "T3": "第3波"}
DEMO   = ["gender", "age", "grade", "sibling", "volunteer"]

# ---- instrument definitions -------------------------------------------------
# A = Basic Empathy Scale (20)   B = GQ-6 (6)
# C = prosocial inventory (15)   D = parent-child attachment (20)
REVERSE = {"A": [1, 7, 8, 13, 18, 19, 20],
           "B": [3, 6],
           "C": [],
           "D": [5, 6, 9, 10, 13, 14, 15, 16]}
ITEMS   = {"EMP": ("A", range(1, 21)), "GRA": ("B", range(1, 7)),
           "PSB": ("C", range(1, 16)), "ATT": ("D", range(1, 21))}

EMP_AFF = [1, 2, 4, 5, 7, 8, 11, 13, 15, 17, 18]        # affective subscale
EMP_COG = [3, 6, 9, 10, 12, 14, 16, 19, 20]             # cognitive subscale
ATT_MOT = [1, 3, 5, 7, 9, 11, 13, 15, 17, 19]           # mother-referenced
ATT_FAT = [2, 4, 6, 8, 10, 12, 14, 16, 18, 20]          # father-referenced
GRA_POS = [1, 2, 4, 5]
GRA_NEG = [3, 6]

# Facets each construct's parcels must draw from proportionally.
FACETS = {"EMP": [EMP_AFF, EMP_COG],
          "ATT": [ATT_MOT, ATT_FAT],
          "GRA": [GRA_POS, GRA_NEG],
          "PSB": [list(range(1, 16))]}

MIN_COMPLETE = 0.60   # a scale score is computed only if >=60% of items answered


def load_wave(sheet):
    """Read one wave. Row 0 is a section banner, row 1 holds the item labels."""
    raw = pd.read_excel(SRC, sheet_name=sheet, header=None)
    header = raw.iloc[1].tolist()
    data   = raw.iloc[2:].reset_index(drop=True)

    cols = ["ID"] + DEMO
    for h in header[6:]:
        h = str(h)
        cols.append("DROP" if h == "nan" else h.split(".")[0])
    data.columns = cols[:data.shape[1]]
    data = data.loc[:, pd.Index(data.columns) != "DROP"]

    data["ID"] = data["ID"].astype(str).str.strip()
    for c in data.columns:
        if c != "ID":
            data[c] = pd.to_numeric(data[c], errors="coerce")
    return data


def reverse_score(df):
    out = df.copy()
    for prefix, items in REVERSE.items():
        for i in items:
            col = f"{prefix}{i}"
            if col in out.columns:
                out[col] = 6 - out[col]          # five-point scale
    return out


def build_parcels(df_t1, prefix, facets, k=3):
    """
    Domain-representative allocation. Within each facet, items are ordered by
    their item-total correlation at Wave 1 and dealt into the k parcels in
    serpentine (round-robin, alternating direction) order, so that every parcel
    draws proportionally from every facet and parcels are balanced on loading.
    Membership is fixed on Wave 1 and reused unchanged at Waves 2 and 3, which
    is the condition required for longitudinal invariance testing.
    """
    parcels = [[] for _ in range(k)]
    j = 0
    for facet in facets:
        cols  = [f"{prefix}{i}" for i in facet]
        total = df_t1[cols].mean(axis=1)
        r     = np.array([df_t1[c].corr(total) for c in cols])
        for idx in np.argsort(-r):                      # strongest item first
            forward = (j // k) % 2 == 0
            pos = (j % k) if forward else (k - 1 - (j % k))
            parcels[pos].append(cols[idx])
            j += 1
    return parcels


def main():
    waves  = {w: reverse_score(load_wave(sh)) for w, sh in SHEETS.items()}
    parcels = {name: build_parcels(waves["T1"], prefix, FACETS[name])
               for name, (prefix, _) in ITEMS.items()}

    print("Parcel assignment (fixed on Wave 1, applied to all waves):")
    for name, ps in parcels.items():
        for i, p in enumerate(ps, 1):
            print(f"  {name} parcel {i} ({len(p):2d} items): {', '.join(sorted(p, key=lambda x: int(x[1:])))}")

    wide = None
    for w, df in waves.items():
        out = df[["ID"]].copy()
        for name, (prefix, rng) in ITEMS.items():
            cols = [f"{prefix}{i}" for i in rng]
            enough = df[cols].notna().sum(axis=1) >= len(cols) * MIN_COMPLETE
            out[f"{name}_{w}"] = df[cols].mean(axis=1).where(enough)
            for i, pc in enumerate(parcels[name], 1):
                out[f"{name}{i}_{w}"] = df[pc].mean(axis=1)
        out[f"EMPaff_{w}"] = df[[f"A{i}" for i in EMP_AFF]].mean(axis=1)
        out[f"EMPcog_{w}"] = df[[f"A{i}" for i in EMP_COG]].mean(axis=1)
        out[f"ATTm_{w}"]   = df[[f"D{i}" for i in ATT_MOT]].mean(axis=1)
        out[f"ATTf_{w}"]   = df[[f"D{i}" for i in ATT_FAT]].mean(axis=1)
        for c in DEMO:
            out[f"{c}_{w}"] = df[c]
        wide = out if wide is None else wide.merge(out, on="ID", how="outer")

    wide.to_csv("wide_all.csv", index=False)

    linked = set(waves["T1"].ID) & set(waves["T2"].ID) & set(waves["T3"].ID)
    analytic = wide[wide.ID.isin(linked)].copy()
    analytic.to_csv("analytic.csv", index=False)

    print(f"\nRecords per wave: "
          f"{len(waves['T1'])}, {len(waves['T2'])}, {len(waves['T3'])}")
    print(f"Linked across all three waves (analytic sample): {len(analytic)}")


if __name__ == "__main__":
    main()
```

```
02_descriptives_reliability.py
"""
02_descriptives_reliability.py
Cronbach's alpha, McDonald's omega, descriptive statistics, the correlation
matrix, Little's MCAR test, the attrition comparison and Mahalanobis outlier
screening. Produces Table 1, Table 2 and Supplementary Table S3 of the paper.

Input : analytic.csv, wide_all.csv, 总表.xlsx
Output: table1_descriptives_reliability.csv, table2_correlations.csv,
        tableS3_attrition.csv, outliers.csv, mcar_and_outliers.txt
"""
import pandas as pd, numpy as np
from scipy import stats
# The helper functions below are re-declared rather than imported, because a
# module name cannot begin with a digit; this keeps each script runnable alone.

SRC     = "总表.xlsx"
SHEETS  = {"T1": "第1波", "T2": "第2波", "T3": "第3波"}
DEMO    = ["gender", "age", "grade", "sibling", "volunteer"]
REVERSE = {"A": [1, 7, 8, 13, 18, 19, 20], "B": [3, 6], "C": [],
           "D": [5, 6, 9, 10, 13, 14, 15, 16]}
ITEMS   = {"ATT": ("D", 20), "EMP": ("A", 20), "GRA": ("B", 6), "PSB": ("C", 15)}
EMP_AFF = [1, 2, 4, 5, 7, 8, 11, 13, 15, 17, 18]
EMP_COG = [3, 6, 9, 10, 12, 14, 16, 19, 20]
LABEL   = {"ATT": "Parent-child attachment", "EMP": "Empathy",
           "GRA": "Gratitude", "PSB": "Prosocial behaviour"}


def load_wave(sheet):
    raw = pd.read_excel(SRC, sheet_name=sheet, header=None)
    header, data = raw.iloc[1].tolist(), raw.iloc[2:].reset_index(drop=True)
    cols = ["ID"] + DEMO
    for h in header[6:]:
        h = str(h)
        cols.append("DROP" if h == "nan" else h.split(".")[0])
    data.columns = cols[:data.shape[1]]
    data = data.loc[:, pd.Index(data.columns) != "DROP"]
    data["ID"] = data["ID"].astype(str).str.strip()
    for c in data.columns:
        if c != "ID":
            data[c] = pd.to_numeric(data[c], errors="coerce")
    for prefix, items in REVERSE.items():
        for i in items:
            if f"{prefix}{i}" in data.columns:
                data[f"{prefix}{i}"] = 6 - data[f"{prefix}{i}"]
    return data


def cronbach_alpha(X):
    X = X.dropna()
    k = X.shape[1]
    return k / (k - 1) * (1 - X.var(ddof=1).sum() / X.sum(axis=1).var(ddof=1))


def mcdonald_omega(X, n_iter=200):
    """
    Omega from a one-factor solution fitted to the item correlation matrix by
    iterated principal-axis extraction (communalities on the diagonal).
    omega = (sum of unstandardised loadings)^2 /
            [(sum of loadings)^2 + sum of residual variances]
    """
    X = X.dropna()
    Rm = np.corrcoef(X.T)
    w, v = np.linalg.eigh(Rm)
    lam = v[:, -1] * np.sqrt(w[-1])
    if lam.sum() < 0:
        lam = -lam
    for _ in range(n_iter):
        h2 = np.clip(lam ** 2, 0, 0.99)
        Rr = Rm.copy()
        np.fill_diagonal(Rr, h2)
        w, v = np.linalg.eigh(Rr)
        lam = v[:, -1] * np.sqrt(max(w[-1], 1e-9))
        if lam.sum() < 0:
            lam = -lam
    sd = X.std(ddof=1).values
    L  = lam * sd
    e  = np.clip(sd ** 2 - L ** 2, 1e-9, None)
    return L.sum() ** 2 / (L.sum() ** 2 + e.sum())


def little_mcar(X, tol=1e-7, n_iter=500):
    """
    Little's (1988) test. EM estimates of the mean vector and covariance matrix
    are obtained first; the statistic then compares each missingness pattern's
    observed means against those estimates. Returns (d2, df, p, n_patterns).
    """
    Xv = X.values.astype(float)
    n, p = Xv.shape
    mu = np.nanmean(Xv, 0)
    S  = np.nan_to_num(np.ma.cov(np.ma.masked_invalid(Xv), rowvar=False).data) + np.eye(p) * 1e-6

    for _ in range(n_iter):                       # EM
        T1, T2 = np.zeros(p), np.zeros((p, p))
        for i in range(n):
            obs = ~np.isnan(Xv[i]); mis = ~obs
            xi = Xv[i].copy(); C = np.zeros((p, p))
            if mis.sum() and obs.sum():
                Soo = S[np.ix_(obs, obs)]; Smo = S[np.ix_(mis, obs)]
                B = Smo @ np.linalg.pinv(Soo)
                xi[mis] = mu[mis] + B @ (Xv[i][obs] - mu[obs])
                C[np.ix_(mis, mis)] = S[np.ix_(mis, mis)] - B @ Smo.T
            elif obs.sum() == 0:
                xi, C = mu.copy(), S.copy()
            T1 += xi; T2 += np.outer(xi, xi) + C
        mu_n = T1 / n
        S_n  = T2 / n - np.outer(mu_n, mu_n)
        done = np.max(np.abs(mu_n - mu)) < tol and np.max(np.abs(S_n - S)) < tol
        mu, S = mu_n, S_n
        if done:
            break

    pattern = pd.DataFrame(~np.isnan(Xv)).astype(int).astype(str).agg("".join, axis=1)
    d2, df = 0.0, 0
    for key, idx in pd.Series(range(n)).groupby(pattern.values):
        idx = list(idx)
        obs = np.array([c == "1" for c in key])
        if obs.sum() == 0:
            continue
        xbar = np.nanmean(Xv[np.ix_(idx, np.where(obs)[0])], 0)
        Soo  = S[np.ix_(obs, obs)]
        d2 += len(idx) * float((xbar - mu[obs]) @ np.linalg.pinv(Soo) @ (xbar - mu[obs]))
        df += obs.sum()
    df -= p
    return d2, int(df), float(1 - stats.chi2.cdf(d2, df)), int(pattern.nunique())


def main():
    waves = {w: load_wave(sh) for w, sh in SHEETS.items()}
    wide  = pd.read_csv("wide_all.csv", dtype={"ID": str})
    ana   = pd.read_csv("analytic.csv", dtype={"ID": str})
    V     = [f"{c}_{w}" for c in ["ATT", "EMP", "GRA", "PSB"] for w in ["T1", "T2", "T3"]]
    log   = []

    # ---- Table 1: descriptives + reliability ------------------------------
    rows = []
    for c in ["ATT", "EMP", "GRA", "PSB"]:
        prefix, n_items = ITEMS[c]
        for w in ["T1", "T2", "T3"]:
            cols = [f"{prefix}{i}" for i in range(1, n_items + 1)]
            s = wide[f"{c}_{w}"]
            rows.append({"Variable": f"{LABEL[c]} {w.replace('T','W')}",
                         "n": int(s.notna().sum()),
                         "M": round(s.mean(), 3), "SD": round(s.std(), 3),
                         "Skewness": round(s.skew(), 3), "Kurtosis": round(s.kurt(), 3),
                         "alpha": round(float(cronbach_alpha(waves[w][cols])), 3),
                         "omega": round(float(mcdonald_omega(waves[w][cols])), 3)})
    t1 = pd.DataFrame(rows)
    t1.to_csv("table1_descriptives_reliability.csv", index=False)

    for w in ["T1", "T2", "T3"]:                 # subscale reliabilities (text only)
        for nm, items in [("affective empathy", EMP_AFF), ("cognitive empathy", EMP_COG)]:
            cols = [f"A{i}" for i in items]
            log.append(f"{nm} {w}: alpha={cronbach_alpha(waves[w][cols]):.3f} "
                       f"omega={mcdonald_omega(waves[w][cols]):.3f}")

    # ---- Table 2: correlations --------------------------------------------
    X = wide[V]
    Rm = X.corr().round(3)
    P  = pd.DataFrame(np.ones_like(Rm), index=Rm.index, columns=Rm.columns)
    for a in V:
        for b in V:
            if a != b:
                s = X[[a, b]].dropna()
                P.loc[a, b] = stats.pearsonr(s[a], s[b])[1]
    Rm.to_csv("table2_correlations.csv")
    log.append(f"correlations: {(P.values[np.triu_indices(len(V),1)] < .001).sum()} "
               f"of {len(V)*(len(V)-1)//2} pairs significant at p < .001")

    # ---- Supplementary Table S3: attrition --------------------------------
    retained = wide[V].notna().all(axis=1)
    att = []
    for v in ["ATT_T1", "EMP_T1", "GRA_T1", "PSB_T1"]:
        g1 = wide.loc[retained, v].dropna()
        g0 = wide.loc[~retained, v].dropna()
        t = stats.ttest_ind(g1, g0, equal_var=False)
        d = (g1.mean() - g0.mean()) / np.sqrt((g1.var() + g0.var()) / 2)
        att.append({"Wave 1 variable": LABEL[v.split("_")[0]],
                    "n retained": len(g1), "M retained": round(g1.mean(), 3),
                    "SD retained": round(g1.std(), 3),
                    "n non-retained": len(g0), "M non-retained": round(g0.mean(), 3),
                    "SD non-retained": round(g0.std(), 3),
                    "t": round(t.statistic, 3), "df": round(t.df, 1),
                    "p": round(t.pvalue, 3), "Cohen's d": round(d, 3)})
    pd.DataFrame(att).to_csv("tableS3_attrition.csv", index=False)

    for c in ["gender_T1", "age_T1", "grade_T1", "sibling_T1", "volunteer_T1"]:
        ct = pd.crosstab(wide[c], retained)
        if ct.shape[0] > 1:
            chi2, p, dof, _ = stats.chi2_contingency(ct)
            log.append(f"retention by {c}: chi2({dof})={chi2:.2f}, p={p:.3f}")

    # ---- Little's MCAR -----------------------------------------------------
    d2, df, p, npat = little_mcar(wide[V])
    log.append(f"Little's MCAR: chi2({df}) = {d2:.2f}, p = {p:.4f}, {npat} patterns")

    # ---- Mahalanobis outlier screening ------------------------------------
    Xc  = ana[V].dropna()
    S   = np.cov(Xc.T); mu = Xc.mean().values
    md  = np.array([(r - mu) @ np.linalg.pinv(S) @ (r - mu) for r in Xc.values])
    crit = stats.chi2.ppf(.999, len(V))
    flagged = ana.loc[Xc.index[md > crit], "ID"]
    flagged.to_frame("ID").to_csv("outliers.csv", index=False)
    log.append(f"Mahalanobis: screened n={len(md)}, critical chi2(12) at p<.001 = {crit:.2f}, "
               f"flagged {len(flagged)} ({100*len(flagged)/len(md):.2f}%), max = {md.max():.2f}")
    log.append(f"listwise deletion would drop {len(ana) - len(Xc)} of {len(ana)} "
               f"({100*(len(ana)-len(Xc))/len(ana):.1f}%)")

    with open("mcar_and_outliers.txt", "w") as fh:
        fh.write("\n".join(log))
    print("\n".join(log))


if __name__ == "__main__":
    main()
```

```
03_invariance.R
# 03_invariance.R -------------------------------------------------------------
# Longitudinal measurement invariance (configural / metric / scalar) for each
# of the four constructs. Produces Table 3 of the paper.
#
# Input : analytic.csv        Output: table3_invariance.csv, d.rds
# Model : one latent variable per wave, three parcel indicators each,
#         residual covariances freed for matching parcels across waves,
#         marker-variable identification (first loading = 1, first intercept = 0).

library(lavaan)
set.seed(20260804)

d <- read.csv("analytic.csv", colClasses = c(ID = "character"))
cat("Analytic N =", nrow(d), "\n")
saveRDS(d, "d.rds")          # reused by scripts 04-06

FI <- c("chisq","df","pvalue","cfi","tli","rmsea",
        "rmsea.ci.lower","rmsea.ci.upper","srmr","aic","bic")

# level 0 = configural, 1 = metric (loadings equal), 2 = scalar (+ intercepts equal)
build_invariance <- function(construct, level){
  p <- function(i, w) sprintf("%s%d_T%d", construct, i, w)
  m <- ""
  for (w in 1:3){
    l2 <- if (level >= 1) "L2" else sprintf("f%dL2", w)
    l3 <- if (level >= 1) "L3" else sprintf("f%dL3", w)
    m <- paste0(m, sprintf("F%d =~ 1*%s + %s*%s + %s*%s\n",
                           w, p(1,w), l2, p(2,w), l3, p(3,w)))
  }
  for (w in 1:3){
    i2 <- if (level >= 2) "i2" else sprintf("f%di2", w)
    i3 <- if (level >= 2) "i3" else sprintf("f%di3", w)
    m <- paste0(m, sprintf("%s ~ 0*1\n%s ~ %s*1\n%s ~ %s*1\n",
                           p(1,w), p(2,w), i2, p(3,w), i3))
  }
  m <- paste0(m, "F1 ~ NA*1\nF2 ~ NA*1\nF3 ~ NA*1\n")
  for (j in 1:3)                                  # correlated parcel residuals
    m <- paste0(m, sprintf("%s ~~ %s\n%s ~~ %s\n%s ~~ %s\n",
                           p(j,1), p(j,2), p(j,1), p(j,3), p(j,2), p(j,3)))
  paste0(m, "F1 ~~ F2 + F3\nF2 ~~ F3\n")
}

results <- NULL
for (construct in c("ATT","EMP","GRA","PSB")){
  fits <- lapply(0:2, function(L)
    cfa(build_invariance(construct, L), data = d,
        missing = "fiml", meanstructure = TRUE))
  names(fits) <- c("Configural","Metric","Scalar")

  tab <- do.call(rbind, lapply(names(fits), function(nm){
    fm <- fitMeasures(fits[[nm]], FI)
    setNames(data.frame(construct, nm, t(round(as.numeric(fm), 3))),
             c("Construct","Model", paste0("V", 1:11)))
  }))
  colnames(tab)[3:13] <- c("chisq","df","p","CFI","TLI","RMSEA",
                           "RMSEA.lo","RMSEA.hi","SRMR","AIC","BIC")

  lrt1 <- lavTestLRT(fits$Configural, fits$Metric)
  lrt2 <- lavTestLRT(fits$Metric,     fits$Scalar)
  tab$dchisq <- c(NA, round(lrt1$`Chisq diff`[2], 3), round(lrt2$`Chisq diff`[2], 3))
  tab$ddf    <- c(NA, lrt1$`Df diff`[2],             lrt2$`Df diff`[2])
  tab$dp     <- c(NA, round(lrt1$`Pr(>Chisq)`[2], 3), round(lrt2$`Pr(>Chisq)`[2], 3))
  tab$dCFI   <- c(NA, round(diff(tab$CFI),   3))
  tab$dRMSEA <- c(NA, round(diff(tab$RMSEA), 3))
  tab$dSRMR  <- c(NA, round(diff(tab$SRMR),  3))
  results <- rbind(results, tab)
}

# Decision criteria: |dCFI| <= .010 and dRMSEA <= .015 (Cheung & Rensvold 2002; Chen 2007)
print(results, row.names = FALSE)
write.csv(results, "table3_invariance.csv", row.names = FALSE)
```

```
04_riclpm.R
# 04_riclpm.R -----------------------------------------------------------------
# Random-intercept cross-lagged panel model and, for comparison, the traditional
# cross-lagged panel model on the same composites.
# Produces Table 4, Table 5 and Fig. 1 input.
#
# Input : d.rds (written by 03_invariance.R)
# Output: table4_ri_correlations.csv, table5A_riclpm_paths.csv,
#         table5B_clpm_paths.csv, riclpm_variance.csv, riclpm_fit.csv, clpm_fit.csv

library(lavaan)
set.seed(20260804)

d <- readRDS("d.rds")
V <- c("ATT","EMP","GRA","PSB")      # composites
L <- c("a","e","g","p")              # short labels used in parameter names
FI <- c("chisq","df","pvalue","cfi","tli","rmsea",
        "rmsea.ci.lower","rmsea.ci.upper","srmr","aic","bic")

## ---- RI-CLPM ---------------------------------------------------------------
# Between-person part: one time-invariant random intercept per construct,
# all three loadings fixed to 1.
ri <- paste(sapply(1:4, function(i)
  sprintf("RI%s =~ 1*%s_T1 + 1*%s_T2 + 1*%s_T3", L[i], V[i], V[i], V[i])),
  collapse = "\n")

# Within-person part: one latent deviation per construct per wave.
wf <- paste(sapply(1:4, function(i)
  paste(sapply(1:3, function(t) sprintf("w%s%d =~ 1*%s_T%d", L[i], t, V[i], t)),
        collapse = "\n")), collapse = "\n")

# Observed residual variances fixed to 0: all variance is carried by the
# random intercept and the within-person component.
fix0 <- paste(sapply(1:4, function(i)
  paste(sapply(1:3, function(t) sprintf("%s_T%d ~~ 0*%s_T%d", V[i], t, V[i], t)),
        collapse = "\n")), collapse = "\n")

# Autoregressive and cross-lagged structure among within-person deviations.
cl <- ""
for (t in 2:3) for (i in 1:4){
  predictors <- paste(sapply(1:4, function(j)
    sprintf("%s%d%d%d*w%s%d", L[j], i, j, t, L[j], t - 1)), collapse = " + ")
  cl <- paste0(cl, sprintf("w%s%d ~ %s\n", L[i], t, predictors))
}

cov_w1  <- paste(apply(combn(4, 2), 2, function(k)
  sprintf("w%s1 ~~ w%s1", L[k[1]], L[k[2]])), collapse = "\n")
cov_w23 <- paste(sapply(2:3, function(t) paste(apply(combn(4, 2), 2, function(k)
  sprintf("w%s%d ~~ w%s%d", L[k[1]], t, L[k[2]], t)), collapse = "\n")), collapse = "\n")
cov_ri  <- paste(apply(combn(4, 2), 2, function(k)
  sprintf("RI%s ~~ RI%s", L[k[1]], L[k[2]])), collapse = "\n")
# Random intercepts orthogonal to the within-person components at wave 1.
orth <- paste(sapply(1:4, function(i) paste(sapply(1:4, function(j)
  sprintf("RI%s ~~ 0*w%s1", L[i], L[j])), collapse = "\n")), collapse = "\n")

model_ri <- paste(ri, wf, fix0, cl, cov_w1, cov_w23, cov_ri, orth, sep = "\n")
writeLines(model_ri, "riclpm_model_syntax.txt")

fit_ri <- lavaan(model_ri, data = d, missing = "fiml", meanstructure = TRUE,
                 int.ov.free = TRUE, int.lv.free = FALSE,
                 auto.var = TRUE, auto.cov.lv.x = FALSE, auto.fix.first = FALSE)

cat("\n--- RI-CLPM fit ---\n"); print(round(fitMeasures(fit_ri, FI), 3))
ss <- standardizedSolution(fit_ri, ci = TRUE, level = .95)

write.csv(ss[ss$op == "~", ], "table5A_riclpm_paths.csv", row.names = FALSE)
write.csv(ss[ss$op == "~~" & grepl("^RI", ss$lhs) & ss$lhs != ss$rhs, ],
          "table4_ri_correlations.csv", row.names = FALSE)
write.csv(data.frame(index = FI, value = round(as.numeric(fitMeasures(fit_ri, FI)), 3)),
          "riclpm_fit.csv", row.names = FALSE)

# Variance decomposition: share of total variance held by the random intercept.
pe   <- parameterEstimates(fit_ri)
v_ri <- pe[pe$op == "~~" & pe$lhs == pe$rhs & grepl("^RI",   pe$lhs), c("lhs","est")]
v_w1 <- pe[pe$op == "~~" & pe$lhs == pe$rhs & grepl("^w.1$", pe$lhs), c("lhs","est")]
write.csv(data.frame(construct = v_ri$lhs, var_between = v_ri$est,
                     var_within = v_w1$est,
                     prop_between = v_ri$est / (v_ri$est + v_w1$est)),
          "riclpm_variance.csv", row.names = FALSE)

## ---- Traditional CLPM (comparison) -----------------------------------------
model_cl <- ""
for (t in 2:3) for (i in 1:4){
  predictors <- paste(sapply(1:4, function(j) sprintf("%s_T%d", V[j], t - 1)), collapse = " + ")
  model_cl <- paste0(model_cl, sprintf("%s_T%d ~ %s\n", V[i], t, predictors))
}
model_cl <- paste0(model_cl,
  paste(apply(combn(4, 2), 2, function(k) sprintf("%s_T1 ~~ %s_T1", V[k[1]], V[k[2]])), collapse = "\n"), "\n",
  paste(sapply(2:3, function(t) paste(apply(combn(4, 2), 2, function(k)
    sprintf("%s_T%d ~~ %s_T%d", V[k[1]], t, V[k[2]], t)), collapse = "\n")), collapse = "\n"))

fit_cl <- sem(model_cl, data = d, missing = "fiml")
cat("\n--- Traditional CLPM fit ---\n"); print(round(fitMeasures(fit_cl, FI), 3))
s2 <- standardizedSolution(fit_cl)
write.csv(s2[s2$op == "~", ], "table5B_clpm_paths.csv", row.names = FALSE)
write.csv(data.frame(index = FI, value = round(as.numeric(fitMeasures(fit_cl, FI)), 3)),
          "clpm_fit.csv", row.names = FALSE)

cat("\nAIC advantage of RI-CLPM:",
    round(fitMeasures(fit_cl,"aic") - fitMeasures(fit_ri,"aic"), 1),
    "| BIC advantage:",
    round(fitMeasures(fit_cl,"bic") - fitMeasures(fit_ri,"bic"), 1), "\n")
```

```
05_indirect_effect.R
# 05_indirect_effect.R --------------------------------------------------------
# Three-wave latent sequential indirect-effect model with autoregressive
# controls, and bias-corrected bootstrap inference on the indirect products.
# Produces Table 6, Table 7 and Figs. 2-4 input.
#
# Input : d.rds        Output: table6_structural_paths.csv,
#                              table7_effects.csv, boot_draws_main.csv
#
# NOTE ON THE BOOTSTRAP
# The reported analysis used B = 5,000 resamples. Because each resample refits a
# FIML latent model, the run was executed in seeded chunks and pooled; a single
# seeded loop, as written below, is statistically equivalent. Expect roughly
# 45-60 min on one core, or set N_BOOT lower to check the pipeline first.

library(lavaan)
set.seed(20260804)

d <- readRDS("d.rds")
d$gender <- ifelse(d$gender_T1 == 2, 1, 0)      # 0 = male, 1 = female
d$age    <- d$age_T1

N_BOOT <- 5000
FI <- c("chisq","df","pvalue","cfi","tli","rmsea",
        "rmsea.ci.lower","rmsea.ci.upper","srmr","aic","bic")

# Measurement block for one construct at one wave. Loadings carry the same
# labels across waves, imposing the metric-invariance constraint supported in
# script 03.
meas <- function(construct, wave, latent)
  sprintf("%s =~ 1*%s1_T%d + %sL2*%s2_T%d + %sL3*%s3_T%d",
          latent, construct, wave, construct, construct, wave,
          construct, construct, wave)

# gratitude_wave = 3 -> primary specification (gratitude concurrent with outcome)
# gratitude_wave = 2 -> alternative specification used in 06_robustness.R
build_model <- function(gratitude_wave){
  mm <- paste(meas("ATT", 1, "ATT1"), meas("EMP", 1, "EMP1"), meas("EMP", 2, "EMP2"),
              meas("GRA", 1, "GRA1"), meas("GRA", gratitude_wave, "GRAm"),
              meas("PSB", 1, "PSB1"), meas("PSB", 3, "PSB3"), sep = "\n")

  rc <- ""                                       # correlated parcel residuals
  for (j in 1:3){
    rc <- paste0(rc, sprintf("EMP%d_T1 ~~ EMP%d_T2\n", j, j))
    rc <- paste0(rc, sprintf("GRA%d_T1 ~~ GRA%d_T%d\n", j, j, gratitude_wave))
    rc <- paste0(rc, sprintf("PSB%d_T1 ~~ PSB%d_T3\n", j, j))
  }

  structural <- "
# a1 attachment -> empathy      arE autoregressive empathy
EMP2 ~ a1*ATT1 + arE*EMP1 + gender + age
# d  empathy -> gratitude       a2 attachment -> gratitude    arG autoregressive
GRAm ~ dm*EMP2 + a2*ATT1 + arG*GRA1 + gender + age
# b1 empathy -> prosocial       b2 gratitude -> prosocial     cp direct effect
PSB3 ~ b1*EMP2 + b2*GRAm + cp*ATT1 + arP*PSB1 + gender + age

ATT1 ~~ EMP1 + GRA1 + PSB1 + gender + age
EMP1 ~~ GRA1 + PSB1 + gender + age
GRA1 ~~ PSB1 + gender + age
PSB1 ~~ gender + age
gender ~~ age

ind1   := a1*b1
ind2   := a2*b2
ind3   := a1*dm*b2
totind := ind1 + ind2 + ind3
total  := cp + ind1 + ind2 + ind3
"
  paste(mm, rc, structural, sep = "\n")
}

model <- build_model(3)
writeLines(model, "indirect_effect_model_syntax.txt")

fit <- sem(model, data = d, missing = "fiml", fixed.x = FALSE)
cat("--- Primary latent model fit ---\n"); print(round(fitMeasures(fit, FI), 3))
cat("R-squared:\n"); print(round(inspect(fit, "r2"), 3))

write.csv(parameterEstimates(fit, ci = TRUE),  "med_main_unstd_ml.csv", row.names = FALSE)
write.csv(standardizedSolution(fit, ci = TRUE),"med_main_std_ml.csv",   row.names = FALSE)
write.csv(data.frame(index = FI, value = round(as.numeric(fitMeasures(fit, FI)), 3)),
          "med_main_fit.csv", row.names = FALSE)

## ---- bias-corrected bootstrap ----------------------------------------------
pe   <- parameterEstimates(fit)
keep <- pe$label != "" | pe$op == ":="
nms  <- make.unique(ifelse(pe$label[keep] == "",
                           paste0(pe$lhs[keep], pe$op[keep], pe$rhs[keep]),
                           pe$label[keep]))

draws <- matrix(NA_real_, N_BOOT, sum(keep)); colnames(draws) <- nms
n <- nrow(d); ok <- 0; attempts <- 0
t0 <- Sys.time()
while (ok < N_BOOT && attempts < N_BOOT * 2){
  attempts <- attempts + 1
  fb <- tryCatch(
    sem(model, data = d[sample(n, n, replace = TRUE), ], missing = "fiml",
        fixed.x = FALSE, start = fit,        # warm start keeps refits fast
        se = "none", test = "none", baseline = FALSE, h1 = FALSE,
        check.gradient = FALSE),
    error = function(e) NULL)
  if (!is.null(fb) && lavInspect(fb, "converged")){
    p <- parameterEstimates(fb); ok <- ok + 1
    draws[ok, ] <- p$est[p$label != "" | p$op == ":="]
  }
  if (ok %% 250 == 0 && ok > 0) cat("  resamples:", ok, "/", N_BOOT, "\n")
}
cat("converged resamples:", ok, "of", attempts, "attempts in",
    round(difftime(Sys.time(), t0, units = "mins"), 1), "min\n")
draws <- draws[1:ok, , drop = FALSE]
write.csv(as.data.frame(draws), "boot_draws_main.csv", row.names = FALSE)

# Bias-corrected percentile interval (Efron 1987):
#   z0     = Phi^-1( proportion of bootstrap estimates below the point estimate )
#   alpha1 = Phi( 2*z0 + z_.025 ),  alpha2 = Phi( 2*z0 + z_.975 )
bc_interval <- function(b, est, level = .95){
  b  <- b[!is.na(b)]
  p0 <- min(max(mean(b < est), 1e-6), 1 - 1e-6)
  z0 <- qnorm(p0)
  a  <- (1 - level) / 2
  c(quantile(b, pnorm(2 * z0 + qnorm(a)),     names = FALSE),
    quantile(b, pnorm(2 * z0 + qnorm(1 - a)), names = FALSE))
}

out <- do.call(rbind, lapply(seq_along(nms), function(i){
  lab <- nms[i]
  est <- pe$est[keep][i]
  bc  <- bc_interval(draws[, i], est)
  pct <- quantile(draws[, i], c(.025, .975), na.rm = TRUE, names = FALSE)
  data.frame(label = lab, est = round(est, 4),
             boot_se = round(sd(draws[, i], na.rm = TRUE), 4),
             bc_lo = round(bc[1], 4), bc_hi = round(bc[2], 4),
             pct_lo = round(pct[1], 4), pct_hi = round(pct[2], 4),
             excludes_zero = as.integer(bc[1] * bc[2] > 0))
}))
print(out, row.names = FALSE)
write.csv(out, "table7_effects.csv", row.names = FALSE)
write.csv(out[out$label %in% c("a1","arE","dm","a2","arG","b1","b2","cp","arP"), ],
          "table6_structural_paths.csv", row.names = FALSE)
```

```
06_robustness.R
# 06_robustness.R -------------------------------------------------------------
# Two prespecified robustness analyses reported in Section 3.5 / Table 8.
#   (a) alternative temporal placement: gratitude at Wave 2 instead of Wave 3,
#       so the second intervening construct precedes the outcome.
#       Estimated with maximum-likelihood intervals, not bootstrapped.
#   (b) outlier-excluded: the 26 multivariate outliers from 02_... removed,
#       primary model refitted with 2,100 bias-corrected bootstrap resamples.
#
# Input : d.rds, outliers.csv    Output: table8_* csv files

library(lavaan)
set.seed(20260804)

d <- readRDS("d.rds")
d$gender <- ifelse(d$gender_T1 == 2, 1, 0)
d$age    <- d$age_T1

FI <- c("chisq","df","pvalue","cfi","tli","rmsea",
        "rmsea.ci.lower","rmsea.ci.upper","srmr")

source_model <- function(){
  # build_model() is defined identically in 05_indirect_effect.R; it is repeated
  # here so this script can be run on its own.
  meas <- function(construct, wave, latent)
    sprintf("%s =~ 1*%s1_T%d + %sL2*%s2_T%d + %sL3*%s3_T%d",
            latent, construct, wave, construct, construct, wave,
            construct, construct, wave)
  function(gratitude_wave){
    mm <- paste(meas("ATT",1,"ATT1"), meas("EMP",1,"EMP1"), meas("EMP",2,"EMP2"),
                meas("GRA",1,"GRA1"), meas("GRA",gratitude_wave,"GRAm"),
                meas("PSB",1,"PSB1"), meas("PSB",3,"PSB3"), sep = "\n")
    rc <- ""
    for (j in 1:3){
      rc <- paste0(rc, sprintf("EMP%d_T1 ~~ EMP%d_T2\n", j, j))
      rc <- paste0(rc, sprintf("GRA%d_T1 ~~ GRA%d_T%d\n", j, j, gratitude_wave))
      rc <- paste0(rc, sprintf("PSB%d_T1 ~~ PSB%d_T3\n", j, j))
    }
    structural <- "
EMP2 ~ a1*ATT1 + arE*EMP1 + gender + age
GRAm ~ dm*EMP2 + a2*ATT1 + arG*GRA1 + gender + age
PSB3 ~ b1*EMP2 + b2*GRAm + cp*ATT1 + arP*PSB1 + gender + age
ATT1 ~~ EMP1 + GRA1 + PSB1 + gender + age
EMP1 ~~ GRA1 + PSB1 + gender + age
GRA1 ~~ PSB1 + gender + age
PSB1 ~~ gender + age
gender ~~ age
ind1   := a1*b1
ind2   := a2*b2
ind3   := a1*dm*b2
totind := ind1 + ind2 + ind3
total  := cp + ind1 + ind2 + ind3
"
    paste(mm, rc, structural, sep = "\n")
  }
}
build_model <- source_model()

## ---- (a) alternative temporal placement: gratitude at Wave 2 ---------------
fit_alt <- sem(build_model(2), data = d, missing = "fiml", fixed.x = FALSE)
cat("--- Alternative specification (gratitude at Wave 2) ---\n")
print(round(fitMeasures(fit_alt, FI), 3))
pe_alt <- parameterEstimates(fit_alt, ci = TRUE)
write.csv(pe_alt[pe_alt$label != "" | pe_alt$op == ":=", ],
          "table8_alternative_unstd.csv", row.names = FALSE)
write.csv(standardizedSolution(fit_alt, ci = TRUE),
          "table8_alternative_std.csv", row.names = FALSE)
write.csv(data.frame(index = FI, value = round(as.numeric(fitMeasures(fit_alt, FI)), 3)),
          "table8_alternative_fit.csv", row.names = FALSE)

## ---- (b) outlier-excluded refit --------------------------------------------
outliers <- read.csv("outliers.csv", colClasses = "character")
# Identifiers are zero-padded ("008"), so both sides are compared as characters.
# The stopifnot() guard fails loudly if a formatting mismatch ever silently
# reduces the number of excluded cases.
d_sens <- d[!(as.character(d$ID) %in% outliers$ID), ]
stopifnot(nrow(d) - nrow(d_sens) == nrow(outliers))
cat("\n--- Outlier-excluded specification: N =", nrow(d_sens),
    "(", nrow(d) - nrow(d_sens), "removed ) ---\n")

model   <- build_model(3)
fit_sen <- sem(model, data = d_sens, missing = "fiml", fixed.x = FALSE)
print(round(fitMeasures(fit_sen, FI), 3))
pe <- parameterEstimates(fit_sen); keep <- pe$label != "" | pe$op == ":="
write.csv(pe[keep, ], "table8_outlier_excluded_unstd.csv", row.names = FALSE)
write.csv(data.frame(index = FI, value = round(as.numeric(fitMeasures(fit_sen, FI)), 3)),
          "table8_outlier_excluded_fit.csv", row.names = FALSE)

N_BOOT <- 2100
nms <- make.unique(ifelse(pe$label[keep] == "",
                          paste0(pe$lhs[keep], pe$op[keep], pe$rhs[keep]),
                          pe$label[keep]))
draws <- matrix(NA_real_, N_BOOT, sum(keep)); colnames(draws) <- nms
n <- nrow(d_sens); ok <- 0
while (ok < N_BOOT){
  fb <- tryCatch(
    sem(model, data = d_sens[sample(n, n, replace = TRUE), ], missing = "fiml",
        fixed.x = FALSE, start = fit_sen, se = "none", test = "none",
        baseline = FALSE, h1 = FALSE, check.gradient = FALSE),
    error = function(e) NULL)
  if (!is.null(fb) && lavInspect(fb, "converged")){
    p <- parameterEstimates(fb); ok <- ok + 1
    draws[ok, ] <- p$est[p$label != "" | p$op == ":="]
  }
}

bc_interval <- function(b, est, level = .95){
  b  <- b[!is.na(b)]
  z0 <- qnorm(min(max(mean(b < est), 1e-6), 1 - 1e-6))
  a  <- (1 - level) / 2
  c(quantile(b, pnorm(2 * z0 + qnorm(a)),     names = FALSE),
    quantile(b, pnorm(2 * z0 + qnorm(1 - a)), names = FALSE))
}
out <- do.call(rbind, lapply(seq_along(nms), function(i){
  bc <- bc_interval(draws[, i], pe$est[keep][i])
  data.frame(label = nms[i], est = round(pe$est[keep][i], 4),
             bc_lo = round(bc[1], 4), bc_hi = round(bc[2], 4),
             excludes_zero = as.integer(bc[1] * bc[2] > 0))
}))
print(out, row.names = FALSE)
write.csv(out, "table8_outlier_excluded_boot.csv", row.names = FALSE)
```

```
07_figures.py
"""
07_figures.py
Generates the four figures reported in the paper, at 300 dpi.

Fig. 1  variance decomposition (between- vs within-person)
Fig. 2  path diagram of the latent sequential indirect-effect model
Fig. 3  forest plot of direct, indirect and total effects
Fig. 4  bootstrap distribution of the sequential indirect association

Input : riclpm_variance.csv, table7_effects.csv, boot_draws_main.csv
Output: fig1_variance.png ... fig4_bootstrap.png
"""
import matplotlib
matplotlib.use("Agg")
import matplotlib.pyplot as plt
import pandas as pd, numpy as np

plt.rcParams.update({"font.family": "DejaVu Sans", "font.size": 9, "axes.linewidth": .8})
DPI = 300

# ---- Fig. 1  variance decomposition ----------------------------------------
v = pd.read_csv("riclpm_variance.csv")
v["name"] = v["construct"].map({"RIa": "Attachment", "RIe": "Empathy",
                                "RIg": "Gratitude",  "RIp": "Prosocial"})
fig, ax = plt.subplots(figsize=(5.4, 3.2))
x = np.arange(len(v)); pct = v["prop_between"].values * 100
ax.bar(x, pct, color="#3b6ea5", width=.6, label="Between-person (random intercept)")
ax.bar(x, 100 - pct, bottom=pct, color="#c8d6e5", width=.6, label="Within-person")
for i, p in enumerate(pct):
    ax.text(i, p / 2, f"{p:.1f}%", ha="center", color="white", fontsize=9, fontweight="bold")
ax.set_xticks(x); ax.set_xticklabels(v["name"]); ax.set_ylabel("Share of total variance (%)")
ax.set_ylim(0, 100); ax.spines[["top", "right"]].set_visible(False)
ax.legend(fontsize=7.5, loc="upper center", bbox_to_anchor=(.5, 1.19), ncol=2, frameon=False)
plt.tight_layout(); plt.savefig("fig1_variance.png", dpi=DPI, bbox_inches="tight"); plt.close()

# ---- Fig. 2  path diagram ---------------------------------------------------
eff = pd.read_csv("table7_effects.csv").set_index("label")
g = lambda k: eff.loc[k, "est"]
fig, ax = plt.subplots(figsize=(9.4, 4.5)); ax.set_xlim(0, 10); ax.set_ylim(0, 5); ax.axis("off")

def box(x, y, w, h, title, sub, fc):
    ax.add_patch(plt.Rectangle((x, y), w, h, fc=fc, ec="#222", lw=1.2, zorder=3))
    ax.text(x + w/2, y + h*.62, title, ha="center", va="center", fontsize=10, fontweight="bold", zorder=4)
    ax.text(x + w/2, y + h*.25, sub,   ha="center", va="center", fontsize=7.8, color="#444", zorder=4)

def arrow(p1, p2, lab, t=.5, dy=.17, dx=0, ls="-"):
    ax.annotate("", xy=p2, xytext=p1, zorder=2,
                arrowprops=dict(arrowstyle="-|>", lw=1.3, color="#111", ls=ls, shrinkA=2, shrinkB=3))
    ax.text(p1[0] + (p2[0]-p1[0])*t + dx, p1[1] + (p2[1]-p1[1])*t + dy, lab,
            ha="center", va="center", fontsize=8.0, zorder=6,
            bbox=dict(fc="white", ec="none", pad=1.5, alpha=.95))

box(0.30, 2.05, 2.0, 1.00, "Attachment", "Wave 1", "#e8eff8")
box(3.35, 3.35, 2.0, 1.00, "Empathy",    "Wave 2", "#e9f4ea")
box(6.35, 3.35, 2.0, 1.00, "Gratitude",  "Wave 3", "#fdf1e3")
box(7.70, 0.75, 2.0, 1.00, "Prosocial behaviour", "Wave 3", "#f7e9f0")
arrow((2.30, 2.80), (3.35, 3.60), f"a\u2081 = {g('a1'):.3f}", .5, .24)
arrow((5.35, 3.85), (6.35, 3.85), f"d = {g('dm'):.3f}",       .5, .24)
arrow((8.35, 3.55), (9.00, 1.75), f"b\u2082 = {g('b2'):.3f}", .45, .10, .62)
arrow((2.30, 3.00), (6.40, 3.55), f"a\u2082 = {g('a2'):.3f}", .86, .26)
arrow((4.20, 3.35), (8.05, 1.75), f"b\u2081 = {g('b1'):.3f}", .30, -.22, 0, (0, (5, 4)))
arrow((2.30, 2.25), (7.70, 1.15), f"c\u2032 = {g('cp'):.3f}", .52, -.22, 0, (0, (5, 4)))
ax.text(5, 0.16, "Unstandardised coefficients. Broken lines mark non-significant paths.",
        ha="center", fontsize=7.6, color="#333")
plt.tight_layout(); plt.savefig("fig2_path_model.png", dpi=DPI, bbox_inches="tight"); plt.close()

# ---- Fig. 3  forest plot ----------------------------------------------------
keys  = ["a1", "dm", "a2", "b1", "b2", "cp", "ind1", "ind2", "ind3", "totind", "total"]
names = {"a1": "Attachment W1 to Empathy W2", "dm": "Empathy W2 to Gratitude W3",
         "a2": "Attachment W1 to Gratitude W3", "b1": "Empathy W2 to Prosocial W3",
         "b2": "Gratitude W3 to Prosocial W3", "cp": "Direct effect (c\u2032)",
         "ind1": "Indirect via empathy only", "ind2": "Indirect via gratitude only",
         "ind3": "Sequential: empathy then gratitude",
         "totind": "Total indirect", "total": "Total effect"}
sub = eff.loc[keys]
fig, ax = plt.subplots(figsize=(6.6, 4.4))
y = np.arange(len(sub))[::-1]
for i, (lab, r) in enumerate(sub.iterrows()):
    col = "#1f4e79" if r["excludes_zero"] else "#9aa5b1"
    ax.plot([r["bc_lo"], r["bc_hi"]], [y[i], y[i]], color=col, lw=2)
    ax.plot(r["est"], y[i], "o", color=col, ms=5.5)
ax.axvline(0, color="#666", lw=.9, ls="--")
ax.set_yticks(y); ax.set_yticklabels([names[k] for k in sub.index], fontsize=8.4)
ax.set_xlabel("Unstandardised estimate with bias-corrected 95% bootstrap CI", fontsize=8.4)
ax.spines[["top", "right"]].set_visible(False)
plt.tight_layout(); plt.savefig("fig3_forest.png", dpi=DPI, bbox_inches="tight"); plt.close()

# ---- Fig. 4  bootstrap distribution of the sequential product ---------------
draws = pd.read_csv("boot_draws_main.csv")
r = eff.loc["ind3"]
fig, ax = plt.subplots(figsize=(5.6, 3.2))
ax.hist(draws["ind3"], bins=70, color="#8fb3d9", edgecolor="white", lw=.3)
ax.axvline(r["est"],   color="#1f4e79", lw=1.6, label=f"Point estimate = {r['est']:.4f}")
ax.axvline(r["bc_lo"], color="#b23a48", lw=1.2, ls="--",
           label=f"BC 95% CI [{r['bc_lo']:.4f}, {r['bc_hi']:.4f}]")
ax.axvline(r["bc_hi"], color="#b23a48", lw=1.2, ls="--")
ax.axvline(0, color="#333", lw=.9, ls=":")
ax.set_xlabel("Sequential indirect association (a$_1$ \u00d7 d \u00d7 b$_2$)")
ax.set_ylabel("Frequency"); ax.legend(fontsize=7.4, frameon=False)
ax.spines[["top", "right"]].set_visible(False)
plt.tight_layout(); plt.savefig("fig4_bootstrap.png", dpi=DPI, bbox_inches="tight"); plt.close()

print("figures written at 300 dpi")
```
